# Prevalence of undernutrition and its associated factors among pregnant women attending antenatal care service in public hospitals in Mogadishu, Somalia

**DOI:** 10.1371/journal.pone.0347187

**Published:** 2026-07-07

**Authors:** Abdullahi Ahmed Tahlil, Naimo Abdikadir Abdillahi, Asho Jama Adam, Anisa Abdi Ali, Fadumo Mohumed Ahmed, Zeynab Osman Abdi, Maryan Muhudin Ali, Muno Abdulle Hubur, Hassan Ibrahim Nor, Mohamed Abdelrahman Mohamed, Liban Abdi Ali, Osman Mohamed Mohamud

**Affiliations:** 1 Ministry of Health and Human Services, Federal Government of Somalia, Mogadishu, Somalia; 2 Center for Epidemic Intelligence, Preparedness and Response, Mogadishu, Somalia; 3 Faculty of Medicine and Health Sciences, Zamzam University of Science and Technology, Mogadishu, Somalia; 4 Department of Nursing and Midwifery, Faculty of Medicine and Health Sciences, Zamzam University of Science and Technology, Mogadishu, Somalia; Federal University of Agriculture and Development Studies, Iragbiji (FUADSI), Nigeria, NIGERIA

## Abstract

**Introduction:**

Maternal undernutrition remains a major public health concern globally, particularly in Somalia, where conflict, poverty, and food insecurity exacerbate nutritional deficiencies. Undernutrition during pregnancy poses serious risks to both maternal and fetal health, including increased maternal mortality, low birth weight, and adverse developmental outcomes. Despite its critical implications, there is limited evidence on the burden and associated factors of maternal undernutrition in Somalia. This study assessed the prevalence and associated factors of undernutrition among pregnant women attending antenatal care (ANC) services in public hospitals in Mogadishu.

**Methods:**

A facility-based cross-sectional study was conducted among 734 pregnant women attending antenatal care (ANC) services in public hospitals in Mogadishu. Participants were selected using a multistage sampling technique. Data were collected using interviewer-administered questionnaires and anthropometric measurements. Undernutrition was assessed using mid-upper arm circumference (MUAC < 23 cm), a measure commonly applied in clinical and humanitarian settings. Household food insecurity was assessed using the Household Food Insecurity Access Scale (HFIAS). Data were analyzed using SPSS version 26, and multivariable logistic regression was performed to identify factors associated with undernutrition at a significance level of p < 0.05.

**Results:**

The prevalence of maternal undernutrition was 75.7% (95% CI: 72.6%–78.9%). Factors independently associated with undernutrition included maternal age 25–31 years (AOR = 2.61, 95% CI: 1.28–5.30) and 32–38 years (AOR = 2.18, 95% CI: 1.04–4.55), tertiary education (AOR = 3.43, 95% CI: 1.89–6.21), employee occupation (AOR = 6.38, 95% CI: 2.90–14.04), private business occupation (AOR = 9.79, 95% CI: 4.27–22.43), large household size (AOR = 1.96, 95% CI: 1.34–2.88), urban residence (AOR = 2.40, 95% CI: 1.39–4.15), household monthly income < USD 500 (AOR = 1.98, 95% CI: 1.33–2.94), lack of latrine facility (AOR = 2.00, 95% CI: 1.23–3.26), second (AOR = 8.59, 95% CI: 5.29–13.97) and third trimester (AOR = 6.91, 95% CI: 2.97–16.08), primigravidity (AOR = 2.29, 95% CI: 1.52–3.45), contraceptive use (AOR = 1.98, 95% CI: 1.34–2.90), substance use (AOR = 2.79, 95% CI: 1.93–4.04), and severe household food insecurity (AOR = 1.76, 95% CI: 1.01–3.08). However, some associations with large effect sizes should be interpreted with caution, as they may reflect residual confounding, small subgroup sizes, or model instability.

**Conclusion:**

This study revealed a high prevalence of maternal undernutrition among pregnant women attending ANC services in Mogadishu, with multiple factors found to be associated with undernutrition. These findings highlight the need for targeted, multi-sectoral interventions to improve maternal nutrition through enhanced food security, health education, and access to nutrition-sensitive and nutrition-specific services. Targeted strategies such as supplementation programs, nutrition counseling, and improved water, sanitation, and hygiene (WASH) services are critical to mitigating maternal undernutrition and achieving Sustainable Development Goals related to maternal and child health.

## Introduction

Undernutrition among pregnant women remains a significant public health challenge worldwide, particularly in low- and middle-income countries such as Somalia, where socioeconomic disparities, limited healthcare access, and food insecurity are prevalent [1]. It is recognized as one of the top ten risk factors contributing to the global burden of disease [2]. The World Health Organization (WHO) defines undernutrition as a condition resulting from inadequate nutrient intake, poor absorption or utilization, or excessive nutrient losses, which lead to harmful health consequences [3]. Undernutrition in pregnant women, characterized by a BMI of less than 18.5 kg/m² and/or a MUAC of less than 23 cm, results from insufficient intake of essential nutrients, leading to low maternal weight gain, anemia, and other deficiencies that increase the risk of adverse outcomes such as preterm birth, low birth weight, and maternal mortality [4,5]. Women are particularly vulnerable to undernutrition due to increased physiological demands during menstruation, pregnancy, childbirth, and lactation [6]. During pregnancy and lactation, women’s nutritional requirements for energy, protein, and micronutrients are significantly elevated to support maternal health and fetal development [7].

Globally, more than one billion adolescent girls and women suffer from undernutrition, including deficiencies in essential micronutrients such as iron, folate, and vitamin A. These deficiencies exacerbate risks during pregnancy and childbirth, contributing to approximately 20 million infants being born annually with low birth weight, an early form of malnutrition; over half of these low-birth-weight births occur in South Asia, and about a quarter in Africa [8,9]. Maternal malnutrition is responsible for approximately 7% of the overall disease burden and contributes to at least a fifth of maternal mortality, with a particularly significant impact on adverse pregnancy outcomes [10]. Maternal undernutrition is associated with intrauterine growth restriction (IUGR), low birth weight, preterm birth, and increased neonatal mortality, accounting for nearly 45% of under-five deaths linked to malnutrition [11]. According to the Global Nutrition Report (2018), approximately 38% of pregnant women globally are anemic, primarily due to iron deficiency, which is a key indicator of undernutrition during pregnancy [12]. Undernutrition is a multifaceted issue influenced by inadequate dietary intake, infectious diseases, poor sanitation, and limited access to healthcare services. These factors are compounded by poverty, food insecurity, lower educational attainment, and cultural practices that influence dietary behaviors during pregnancy [13,14]. These consequences contribute substantially to the global burden of maternal and child morbidity and mortality, particularly in low- and middle-income countries (LMICs), where the prevalence of undernutrition among pregnant women tends to be higher than in developed nations [15].

In Africa, the prevalence of undernutrition among pregnant women remains alarmingly high, with approximately 34.8% of women of reproductive age in sub-Saharan Africa being undernourished, with nutritional deficiencies contributing to nearly 20% of maternal deaths in the region [13,16]. The prevalence among pregnant women is often higher due to compounded factors such as food insecurity, poverty, limited access to quality healthcare, inadequate nutritional education, and sociocultural practices that influence dietary habits [17]. These determinants are further intensified in fragile settings characterized by conflict, displacement, and recurrent emergencies, which disrupt food systems and healthcare delivery.

In Somalia, a country grappling with prolonged conflict, recurrent droughts, and fragile health systems, the maternal mortality rate is one of the highest globally, at 692 deaths per 100,000 live births, with malnutrition being a leading indirect cause [18,19]. According to the Somali Health and Demographic Survey 2020, approximately 15% of pregnant women in Somalia experience undernutrition [20]. It is important to note that national estimates, such as those reported in the Somali Health and Demographic Survey (SHDS 2020), are primarily based on body mass index (BMI), which reflects chronic nutritional status and may not adequately capture acute changes in maternal nutritional status during pregnancy. In contrast, the present study uses mid-upper arm circumference (MUAC), which is more sensitive to acute undernutrition and is widely recommended in clinical and humanitarian settings for its simplicity and reliability. These methodological differences are critical and should be considered when interpreting and comparing prevalence estimates across studies. Similarly, only 31% of pregnant women receive antenatal care (ANC) from skilled providers, and a mere 24% complete the WHO-recommended four ANC visits, limiting early detection and management of nutritional deficiencies [20]. Recent studies in Mogadishu indicate alarming rates of anemia (69%) among pregnant women, driven by factors such as early marriage (<16 years), low household income (<$100/month), inadequate iron supplementation, and poor dietary practices, including infrequent meat consumption and tea intake, which inhibits iron absorption [21]. Additionally, food insecurity affects over 24.5% of households, severely restricting access to diverse, nutrient-rich diets essential for maternal and fetal health [22].

Although the SHDS 2020 reported a high prevalence of malnutrition among pregnant women in Somalia, this estimate was derived from a nationally representative household survey that relied primarily on body mass index (BMI) and did not focus on urban humanitarian settings or facility-based populations. In contrast, the present study specifically assessed acute maternal undernutrition using mid-upper arm circumference (MUAC < 23 cm) among pregnant women attending antenatal care services in public hospitals in Mogadishu, a city characterized by prolonged conflict, mass internal displacement, and severe urban poverty. Pregnant women attending antenatal care services in Mogadishu represent a particularly high-risk subgroup, as many reside in urban poor settings characterized by overcrowding, food insecurity, and limited access to stable income and nutritious foods. Additionally, Mogadishu hosts a large population of internally displaced persons (IDPs), who are disproportionately affected by acute malnutrition and rely heavily on public health facilities for essential maternal care.

Furthermore, existing studies in Somalia have largely concentrated on anemia or child malnutrition, with limited facility-based evidence focusing on maternal undernutrition during pregnancy, particularly using MUAC as a screening tool. MUAC is more sensitive to short-term nutritional stress and is recommended in emergency and humanitarian contexts where rapid assessment is required. Existing studies in Somalia have primarily focused on anemia or child malnutrition. This study, therefore, aims to assess the prevalence of undernutrition and its associated factors among pregnant women attending ANC services at public hospitals in Mogadishu, Somalia. The findings will contribute to the sparse body of evidence on maternal nutrition in Somalia and support policymakers in designing effective strategies to mitigate undernutrition. Addressing this gap is critical to achieving Sustainable Development Goals (SDGs) 2 (Zero Hunger) and 3 (Good Health and Well-being), particularly in a setting where maternal and child survival depend on equitable access to nutrition and healthcare services. This study provides context-specific, clinically relevant evidence that complements national survey findings and addresses an important evidence gap regarding maternal undernutrition in Mogadishu’s urban and displaced populations.

## Methods and materials

### Study design and settings

This study employed a facility-based cross-sectional design to determine the prevalence of undernutrition and its associated factors among pregnant women attending antenatal care (ANC) services. The study was conducted in Mogadishu, Somalia, which is the largest city and the country’s capital. It is located in the coastal region of Banadir in the Horn of Africa and is home to more than 2.7 million residents. Mogadishu is bordered by the regions of Middle Shebelle and Lower Shebelle, as well as the Indian Ocean, and consists of 20 districts.

This study was conducted in selected public hospitals in Mogadishu, including Benadir, Demartino, and SOS hospitals. Benadir Hospital serves as a public teaching hospital, a mother-and-child hospital, and a nationwide referral hospital. It was built in 1976 and has a capacity of approximately 550–700 beds, managed by the Federal Ministry of Health (FMOH). Demartino Hospital is a public referral hospital serving the most vulnerable populations, including pregnant women and children. It was established in 1922 and has a capacity of more than 280 beds. It is also managed by the FMOH. SOS Hospital is a non-governmental hospital administered by SOS Children’s Villages International and focuses on child and maternal health. It was opened in 1989 and has a capacity of approximately 150 beds. This selected hospital serves as the primary point of ANC service delivery for a diverse population of pregnant women in the region. The hospital conducts approximately 1,200 deliveries per month, highlighting its critical role in maternal health in the region.

### Study period and population

Data collection and participant recruitment were carried out from 25/04/2025–29/05/2025. The target population comprised all pregnant women aged 18–49 years attending ANC clinics at the selected public hospitals during the study period. Inclusion criteria included women who were in any trimester of pregnancy, attending routine ANC visits, and willing to participate in the study after providing informed consent. Women with critical medical conditions, severe disabilities, or conditions that could interfere with anthropometric measurements or data collection were excluded to ensure the accuracy and reliability of the data collected.

### Sample size and sampling procedure

Sample size determination was calculated based on the single proportion formula:n = Z^2^ × p×(1-p)/d^2^, where: *n* is the required sample size, *Z* is the Z-score corresponding to the desired 1.96 for a 95% confidence level, *p* is the estimated proportion of the attribute in the population, *q* is 1 − p, and *d* is the margin of error. Considering an anticipated prevalence of undernutrition of 39.2% from previous studies in Ethiopia [23], a 95% confidence level, a 3.7% margin of error, and accounting for a non-response rate of 10%. Using these values in the formula: n = (1.96)^2^ * 0.392 * (1-0.392) / (0.037)^2^ = 670. To account for a potential non-respondent rate of 10%, the final sample size was calculated using the following formula: n / (1 – non-respondents’ rate) = 670 / (1 - 0.1) = 744. Therefore, the final sample size for this study, after accounting for potential non-respondents, was 744 participants.

A multi-stage sampling technique was employed to select participants. First, a list of public hospitals offering antenatal care services in Mogadishu was compiled. From this list, three hospitals were randomly selected. Subsequently, a systematic sampling method was used to select participants. Every *k*^*th*^ eligible woman attending the ANC clinic was approached after a random starting point to ensure fairness and representativeness. The sampling interval (k) was determined by dividing the estimated total number of ANC attendees during the data collection period by the allocated sample size for each hospital. For example, if an estimated 1,200 pregnant women attended ANC services during the study period and the allocated sample size for a given hospital was 300, the sampling interval (k) was calculated as 1,200/300 = 4. In this case, every fourth eligible woman was selected after a random starting point. A random number between 1 and *k* was selected as the starting point, and subsequent participants were chosen by adding *k* to the initial number until the desired sample size was reached, proportionally across the hospitals and shifts.

### Data collection instruments and procedures

Data collection involved two main components: interviewer-administered questionnaires and anthropometric measurements. Data were collected using a structured questionnaire adapted from previous studies [23,24]. The questionnaire was developed in English, then translated into Somali, and subsequently back-translated by a bilingual expert to ensure accuracy. The questionnaire includes sections on socio-demographic characteristics, obstetric history, medical history, Household Food Insecurity Access Scale (HFIAS), 24-hour dietary recall, and anthropometric measurements. Socio-demographic information and obstetric and medical histories captured data on age, education level, occupation, marital status, gravidity, parity, and medical history.

Household food insecurity was assessed using the Household Food Insecurity Access Scale (HFIAS) developed by the Food and Nutrition Technical Assistance (FANTA) project [25]. The HFIAS consists of nine standardized occurrence questions that capture three domains of food insecurity: anxiety about food supply, insufficient food quality, and insufficient food intake over the previous four weeks. Each occurrence question is followed by a frequency-of-occurrence response categorized as rarely (1–2 times), sometimes (3–10 times), or often (>10 times). Households were categorized into four levels: food secure, mildly food insecure, moderately food insecure, and severely food insecure according to standard HFIAS guidelines.

Dietary diversity was assessed using a 24-hour dietary recall method [26]. Participants were asked to recall all foods and beverages consumed in the previous 24 hours, and the reported items were categorized into the ten standard food groups defined by the Minimum Dietary Diversity for Women (MDD-W) indicator. These food groups include: (1) grains, white roots and tubers, and plantains; (2) pulses (beans, peas, and lentils); (3) nuts and seeds; (4) dairy products; (5) meat, poultry, and fish; (6) eggs; (7) dark green leafy vegetables; (8) vitamin A-rich fruits and vegetables; (9) other vegetables; and (10) other fruits. A Dietary Diversity Score (DDS) was calculated for each participant by summing the number of food groups consumed, with scores ranging from 0 to 10. Following established guidelines, women who consumed foods from more than five food groups were classified as having adequate dietary diversity, while those consuming five and fewer food groups were considered to have inadequate dietary diversity [27]. This approach provides a standardized proxy measure of micronutrient adequacy and distinguishes dietary diversity (the number of food groups consumed) from food variety (the number of individual food items consumed), thereby ensuring a more accurate assessment of overall diet quality among pregnant women.

Anthropometric measurements were conducted following WHO guidelines. Height was measured using a portable stadiometer to the nearest 0.1 cm, with participants standing upright without shoes, heels together, and looking straight ahead. Weight was measured using a calibrated digital scale to the nearest 0.1 kg, with participants wearing light clothing and without shoes. Mid-Upper Arm Circumference (MUAC) was measured using a non-stretchable measuring tape. The measurement was taken at the midpoint between the acromion process and the olecranon process on the left arm, with the arm relaxed and hanging freely. The tape was snug but did not compress the tissues. The measurement was recorded to the nearest 0.1 cm. Undernutrition was defined using a MUAC cutoff of <23 cm, consistent with World Health Organization (WHO) and emergency nutrition guidelines, which recommend this threshold for identifying maternal undernutrition in resource-limited and humanitarian settings [28]. MUAC is considered a practical and reliable indicator of acute nutritional status during pregnancy, particularly in contexts where body mass index (BMI) measurement may be less feasible or reliable.

All measurements were performed twice, and the average value was used for analysis to enhance reliability. Measurements were conducted in a private setting to ensure confidentiality and participant comfort. Data collection was carried out by seven trained research assistants at selected public hospitals. Prior to data collection, the research assistants received training in administering questionnaires, taking anthropometric measurements, and ensuring ethical treatment of participants. Each participant was informed about the study’s purpose and procedures, and written informed consent was obtained before data collection.

### Data quality control

To ensure data quality, the questionnaire was pre-tested on 5% of the sample size in a comparable setting outside the study area, and necessary modifications were made. Data collectors received a two-day training on study objectives, interview techniques, anthropometric measurements, and ethical considerations. Supervisors regularly monitored data collection processes, checked the completeness and consistency of the data, and provided ongoing support. Double data entry was performed to minimize entry errors, and data cleaning was conducted before analysis.

### Data analysis

Data were entered and cleaned into IBM SPSS Version 26. Descriptive statistics, including frequencies, percentages, means, and standard deviations, were used to summarize the sociodemographic characteristics and nutritional status of the participants. The prevalence of undernutrition was determined based on MUAC thresholds as per WHO criteria. Bivariate analysis was conducted to identify potential factors associated with undernutrition using chi-square tests. Variables were selected for inclusion in the multivariable logistic regression model based on both statistical and theoretical considerations. Specifically, variables with a p-value <0.25 in the bivariate analysis were included, along with variables identified as important predictors of undernutrition. The use of a p-value threshold of <0.25 at the bivariate stage is a commonly applied approach to ensure that potentially important variables are not prematurely excluded from the multivariable model. The model’s goodness-of-fit was evaluated using the Hosmer-Lemeshow test. Multicollinearity among independent variables was assessed using variance inflation factors (VIF), with a VIF value greater than 10 considered indicative of significant multicollinearity. No variables exceeded this threshold, and all were retained in the final model. Adjusted odds ratios (AORs) with 95% confidence intervals (CI) were reported, and statistical significance was set at p < 0.05.

### Ethics approval and consent to participate

This study was conducted in accordance with the Declaration of Helsinki and was approved by the Institutional Review Board (IRB) of the Research and Innovation Center of Zamzam University of Science and Technology [Ref: ZUST/RIC/IRB/008/April/2025]. Permission to conduct the study was secured from the administrative authorities of the selected hospitals. Written informed consent was obtained from all participants after they were fully informed about the study objectives, procedures, potential risks, and benefits. Confidentiality and anonymity were strictly maintained throughout the study. Participation was entirely voluntary, and respondents were assured of their right to withdraw at any stage without repercussions.

## Results

### Socio-demographic characteristics of the participants

Out of the eligible participants, a total of 734 pregnant women successfully participated in the study, yielding a response rate of 98.7%. The mean age of the participants was 29.3 ± 6.7 years, ranging from 18 to 47 years. The majority, 259 (35.3%), were aged 25–31 years. Most participants, 487 (66.3%), were married. More than half, 398 (54.2%), had no formal education. Regarding occupation, 457 (62.3%) were housewives. A larger proportion, 436 (59.4%), belonged to households with more than seven members. The majority, 591 (80.5%), resided in urban areas, and 470 (64.0%) reported a household monthly income of less than 500 USD. More than half, 447 (60.9%), had at least one child under five years old in the household. Most households, 606 (82.6%), had access to safe drinking water, and 653 (89.0%) had latrine facilities. (Table 1)

**Table 1:** Socio-demographic Characteristics of Pregnant Women Attending Antenatal Care in Public Hospitals in Mogadishu, Somalia (n = 734).

Variables	Frequency	Percentage
**Age (In Years)**		
18–24	183	24.9
25–31	259	35.3
32–38	195	26.6
≥ 39	97	13.2
**Marital Status**		
Married	487	66.3
Widowed	64	8.7
Divorced	183	24.9
**Educational Level**		
No formal	398	54.2
Primary	176	24.0
Secondary	107	14.6
Tertiary	53	7.2
**Occupation**		
Daily Laborer	85	11.6
Employee	115	15.7
Housewife	457	62.3
Private Business	77	10.5
**Family Size**		
≤ 7	298	40.6
> 7	436	59.4
**Residence**		
Rural	143	19.5
Urban	591	80.5
**Household Monthly Income (In USD)**		
< 500	470	64.0
≥ 500	264	36.0
**Presence of under-five children in the Household**		
No	287	39.1
Yes	447	60.9
**Source of Drinking Water**		
Safe	606	82.6
Unsafe	128	17.4
**Availability of the Latrine facility**		
No	81	11.0
Yes	653	89.0

### Reproductive and medical characteristics of the participants

More than half of the participants, 380 (51.8%), were married before the age of 18. Most women, 616 (83.9%), were in their first trimester of pregnancy at the time of the survey. The majority were multigravida, 542 (73.8%), and multiparous, 658 (89.6%). More than half, 430 (58.6%), had a birth interval of ≤2 years, while 600 (81.7%) reported that the current pregnancy was intended. Regarding antenatal care (ANC), 288 (39.2%) attended two visits, while only 86 (11.7%) had four or more visits. About 471 (64.2%) of the participants reported previous contraceptive use, and 447 (60.9%) received nutritional advice during ANC. More than half reported iron and folic acid supplementation, 402 (54.8%), and deworming, 400 (54.5%). A significant proportion, 451 (61.4%), had a history of previous pregnancy-related complications, while 426 (58.0%) reported current illness. Additionally, 403 (54.9%) experienced frequent illness, and 352 (48.0%) had a chronic illness. About one-quarter, 196 (26.7%), admitted to substance use (Table 2).

**Table 2:** Reproductive and Medical Characteristics of Pregnant Women Attending Antenatal Care in Public Hospitals in Mogadishu, Somalia (n = 734).

Variables	Frequency	Percentage
**Age at first marriage**		
< 18	380	51.8
≥ 18	354	48.2
**Trimester of pregnancy**		
First	616	83.9
Second	93	12.7
Third	25	3.4
**Gravida (Number of Pregnancies)**		
Primigravida	192	26.2
Multigravida	542	73.8
**Parity (Number of Births)**		
Primiparous	76	10.4
Multiparous	658	89.6
**Birth Interval**		
≤ 2 years	430	58.6
> 2 years	304	41.4
**Intention of the current pregnancy**		
No	134	18.3
Yes	600	81.7
**Number of ANC Visits**		
1 time	229	31.2
2 times	288	39.2
3 times	131	17.8
≥ 4 times	86	11.7
**Previous Contraceptive Use**		
No	263	35.8
Yes	471	64.2
**Nutrition Advice during a Pregnancy Visit**		
No	287	39.1
Yes	447	60.9
**Use of Iron and Folic Acid Supplementation**		
No	332	45.2
Yes	402	54.8
**Deworming**		
No	334	45.5
Yes	400	54.5
**History of Previous Complications**		
No	283	38.6
Yes	451	61.4
**History of Current Illness**		
No	308	42.0
Yes	426	58.0
**History of Frequent Illness**		
No	331	45.1
Yes	403	54.9
**History of Chronic Illness**		
No	382	52.0
Yes	352	48.0
**Substance Use**		
No	538	73.3
Yes	196	26.7

### Household Food Insecurity Access Scale (HFIAS) of the respondents

**Fig 1:**
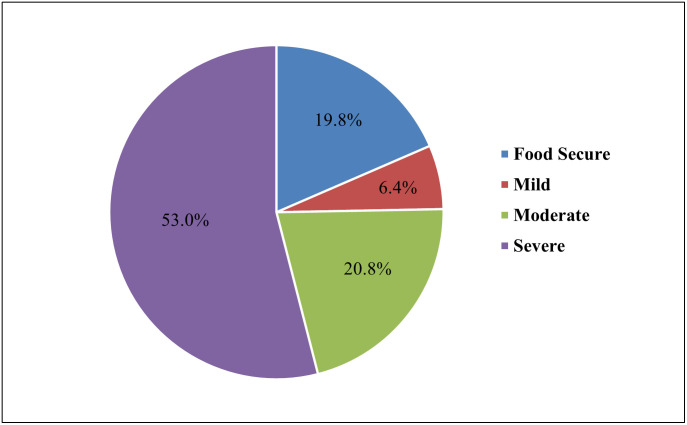
Household food insecurity status among pregnant women attending antenatal care in a public hospital in Mogadishu, Somalia (n = 734).

The findings revealed a high prevalence of household food insecurity among the study participants. The findings showed that only 145 (19.8%) were food secure. More than half, 389 (53.0%), of the households were classified as severely food insecure, while 153 (20.8%) were moderately insecure, and 47 (6.4%) were mildly insecure (Fig 1). More than half of the participants, 474 (64.6%), reported experiencing worry about insufficient food during the four weeks preceding the survey. Similarly, 468 (63.8%) were unable to consume their preferred foods due to limited resources, while 447 (60.9%) indicated reduced dietary variety for the same reason. Furthermore, a considerable proportion of households consumed less preferred or undesirable foods, 450 (61.3%), and experienced reductions in meal size, 462 (62.9%), and frequency of daily meals, 463 (63.1%), because of food scarcity. Alarmingly, 453 (61.7%) of participants reported going to bed hungry, and 462 (62.9%) experienced going a whole day and night without food (Table 3).

**Table 3:** Household Food Insecurity Access Scale (HFIAS) among Households Attending Antenatal Care in Public Hospitals in Mogadishu, Somalia (n = 734).

Variables	No (%)	Yes (%)
Household experienced worry about food being insufficient in the past 4 weeks	260 (35.4)	474 (64.6)
Household members are unable to consume preferred foods due to resource constraints	290 (39.5)	444 (60.5)
Household members are unable to consume the desired foods because of a lack of resources	266 (36.2)	468 (63.8)
Limited dietary variety due to a lack of resources	287 (39.1)	447 (60.9)
Consumption of foods that are less preferred or undesirable due to resource limitations	284 (38.7)	450 (61.3)
Reduction in meal size due to food scarcity	272 (37.1)	462 (62.9)
A decrease in the number of meals per day due to insufficient food	271 (36.9)	463 (63.1)
Going to sleep hungry due to food shortages	281 (38.3)	453 (61.7)

### Dietary characteristics and dietary diversity of the participants

**Fig 2:**
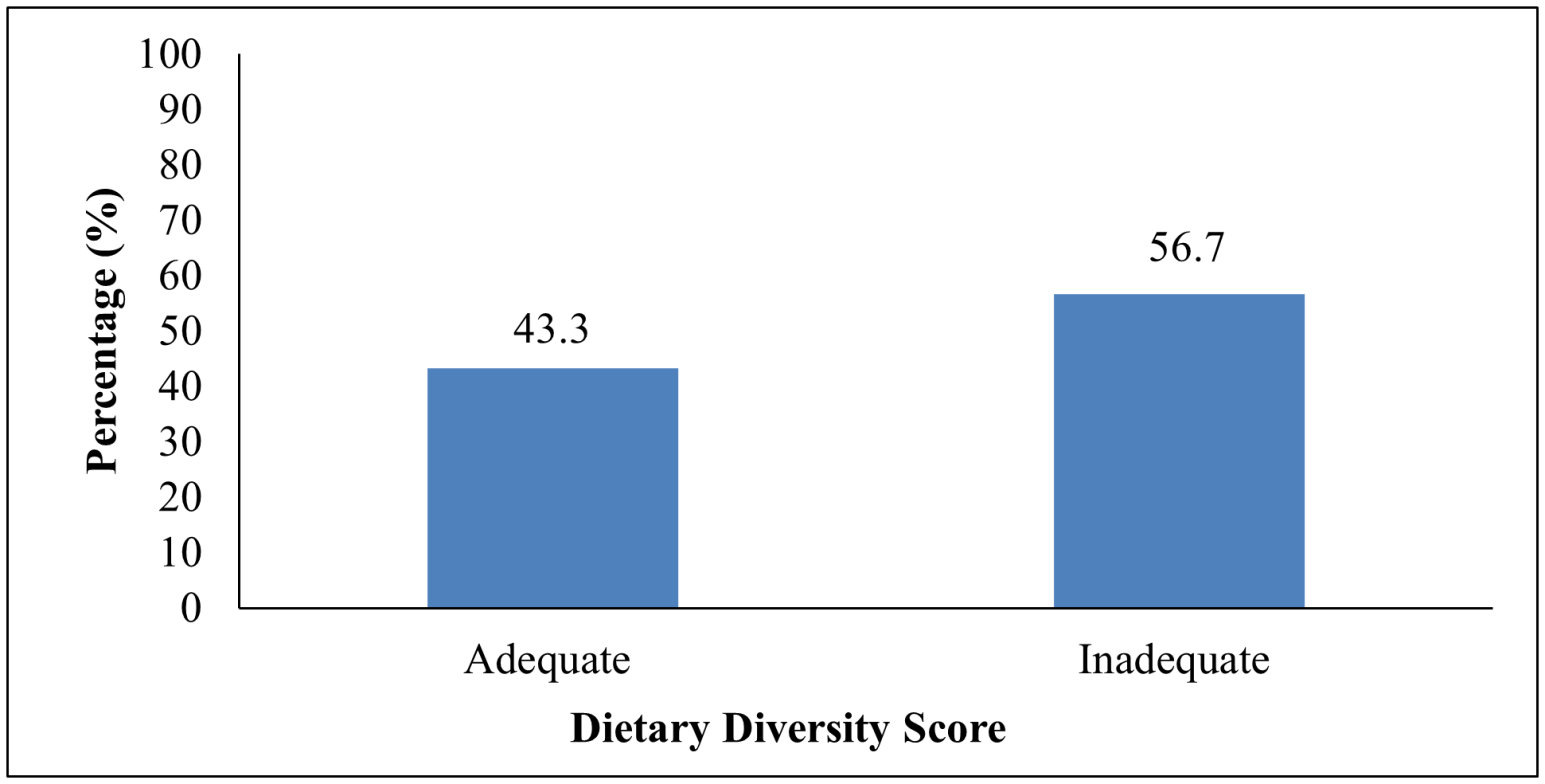
Dietary Diversity Status of Pregnant Women Attending Antenatal Care in Public Hospitals, Mogadishu, Somalia (n = 734).

Consumption of individual food groups was assessed using binary responses to describe dietary patterns, while overall dietary adequacy was determined using the Dietary Diversity Score (DDS) based on the Minimum Dietary Diversity for Women (MDD-W) indicator. The findings revealed that 416 (56.7%) of the participants had inadequate dietary diversity, while 318 (43.3%) achieved adequate dietary diversity (Fig 2), indicating that the majority did not meet the minimum recommended consumption of at least five food groups. The results showed that a substantial proportion of participants did not consume several nutrient-rich food groups, including grains, white roots, tubers, and plantains (414, 56.4%), nuts and seeds (404, 55.0%), meat, poultry, and fish (410, 55.9%), eggs (441, 60.1%), dark green leafy vegetables (421, 57.4%), vitamin A-rich foods (400, 54.5%), other vegetables (481, 65.5%), and other fruits (444, 60.5%). In contrast, relatively higher proportions reported consuming pulses (412, 56.1%) and dairy products (385, 52.5%) (Table 4)

**Table 4:** Consumption of Individual Food Groups Among Pregnant Women Attending Antenatal Care in Public Hospitals in Mogadishu, Somalia (n = 734).

Variables	No (%)	Yes (%)
Grains, white roots, tubers, and plantains	414 (56.4)	320 (43.6)
Pulses	322 (43.9)	412 (56.1)
Nuts and seeds	404 (55.0)	330 (45.0)
Dairy products	349 (47.5)	385 (52.5)
Meat, poultry, and fish	410 (55.9)	324 (44.1)
Eggs	441 (60.1)	293 (39.9)
Dark green leafy vegetables	421 (57.4)	313 (42.6)
Vitamin A-rich foods	400 (54.5)	334 (45.5)
Other vegetables	481 (65.5)	253 (34.5)
Other fruits	444 (60.5)	290 (39.5)

### Nutritional status of participants

**Fig 3:**
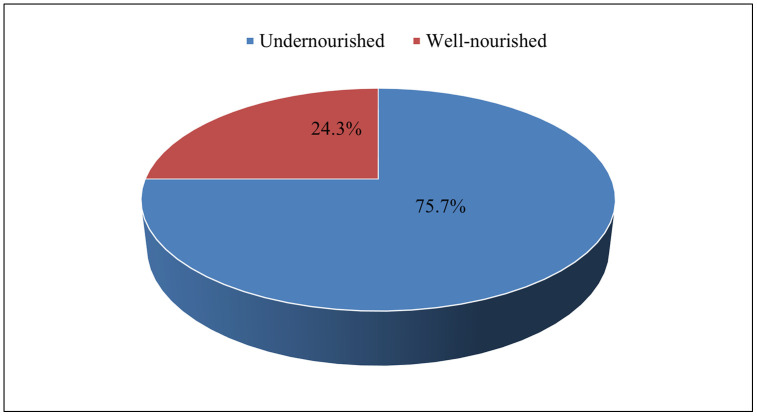
Prevalence of undernutrition among pregnant women attending antenatal care in Public Hospitals in Mogadishu, Somalia (n = 734).

In this study, the prevalence of undernutrition (MUAC < 23 cm) among pregnant women attending antenatal care services in public hospitals in Mogadishu was found to be 75.7% (95% CI: 72.6%–78.9%) (Fig 3). The mean MUAC of participants was 21.1 ± 2.6 cm, indicating a generally low average nutritional status within the study population.

### Factors associated with undernutrition

Table 5 presents the results of a multivariable logistic regression analysis identifying factors independently associated with undernutrition (MUAC < 23 cm) among pregnant women attending antenatal care in public hospitals in Mogadishu, Somalia. The study identified maternal age, educational level, occupation, household size, residence, income, sanitation access, gestational age, gravidity, contraceptive use, and household food insecurity as significant predictors of maternal undernutrition. The findings are reported as adjusted odds ratios (AOR) with 95% confidence intervals, accounting for potential confounding factors. Statistically significant associations (p < 0.05) are noted.

**Table 5:** Multivariable analysis of factors associated with undernutrition among pregnant women attending antenatal care in Public Hospitals in Mogadishu, Somalia (n = 734).

Variables	Undernutrition(MUAC < 23 cm)		COR (95%CI)	AOR (95%CI)	P Value
	Yes	No			
**Age (In Years)**					
18–24	150	33	1.30 (0.66-2.58)	1.31 (0.61-2.84)	0.491
25–31	180	79	2.60 (1.39-4.86)	2.61 (1.28-5.30)	0.008
32–38	143	52	2.16 (1.13-4.13)	2.18 (1.04-4.55)	0.038
≥ 39	83	14	1		
**Educational Level**					
No formal	311	87	1		
Primary	139	37	0.95 (0.62-1.47)	1.21 (0.74-2.00)	0.450
Secondary	79	28	1.27 (0.77-2.07)	1.20 (0.73-1.98)	0.467
Tertiary	27	26	3.44 (1.91-6.20)	3.43 (1.89-6.21)	<0.001
**Occupation**					
Daily Laborer	76	9	1		
Employee	65	50	6.50 (2.97-14.22)	6.38 (2.90-14.04)	<0.001
Housewife	380	77	1.71 (0.82-3.56)	1.80 (0.86-3.77)	0.118
Private Business	35	42	10.13 (4.45-23.10)	9.79 (4.27-22.43)	<0.001
**Family Size**					
≤ 7	251	47	1	1	
> 7	305	131	2.29 (1.58-3.33)	1.96 (1.34-2.88)	0.001
**Residence**					
Rural	126	17	1	1	
Urban	430	161	2.78 (1.62-4.75)	2.40 (1.39-4.15)	0.002
**HH Monthly Income (In USD)**					
< 500	333	137	2.24 (1.52-3.30)	1.98 (1.33-2.94)	0.001
≥ 500	223	41	1	1	
**Presence of U-5 children in the HH**					
No	225	62	1	1	
Yes	331	116	1.27 (0.90-1.81)	1.27 (0.89-1.80)	0.192
**Availability of the Latrine facility**					
No	51	30	2.01 (1.23-3.27)	2.00 (1.23-3.26)	0.005
Yes	505	148	1	1	
**Age at first marriage**					
< 18	302	78	1.52 (1.09-2.14)	1.28 (0.88-1.85)	0.201
≥ 18	254	100	1		
**Trimester of pregnancy**					
First	508	108	1		
Second	37	56	7.12 (4.48-11.33)	8.59 (5.29-13.97)	<0.001
Third	11	14	5.99 (2.65-13.55)	6.91 (2.97-16.08)	<0.001
**Number of Pregnancies**					
Primigravida	133	59	1.578 (1.09-2.28)	2.29 (1.52-3.45)	<0.001
Multigravida	423	119	1		
**Previous Contraceptive Use**					
No	220	43	1		
Yes	336	135	2.06 (1.40-3.02)	1.98 (1.34-2.90)	0.001
**History of Previous Complications**					
No	201	82	1		
Yes	355	96	0.66 (0.47-0.93)	0.74 (0.49-1.11)	0.145
**History of Frequent Illness**					
No	238	93	1		
Yes	318	85	0.68 (0.49-0.96)	0.94 (0.62-1.42)	0.761
**History of Chronic Illness**					
No	306	76	1	1	
Yes	250	102	1.64 (1.17-2.31)	1.31 (0.92-1.88)	0.139
**Substance Use**					
No	439	99	1		
Yes	117	79	2.99 (2.09-4.29)	2.79 (1.93-4.04)	<0.001
**Household food insecurity status**					
Food Secure	126	19	1		
Mild	38	9	1.57 (0.66-3.76)	1.37 (0.57-3.31)	0.482
Moderate	112	41	2.43 (1.33-4.43)	1.80 (0.97-3.35)	0.063
Severe	280	109	2.58 (1.52-4.39)	1.76 (1.01-3.08)	0.047

Age demonstrated a significant association, with women aged 25–31 years exhibiting 2.61 times higher odds of undernutrition (AOR = 2.61, 95% CI: 1.28–5.30, p = 0.008), and women aged 32–38 years (AOR = 2.18, 95% CI: 1.04–4.55, p = 0.038) were more likely to be undernourished compared to those aged ≥39 years. Educational attainment was significantly associated with undernutrition. Women with a tertiary education had 3.43 times higher odds of undernutrition (AOR = 3.43, 95% CI: 1.89–6.21, p < 0.001) compared to those with no formal education. However, this estimate should be interpreted with caution, as the number of participants in the tertiary education category was relatively small, which may limit the stability and precision of the observed association. This is further reflected in the relatively wide confidence intervals, suggesting potential variability in the estimate. Maternal occupation was significantly associated with nutritional status. Pregnant women employed in private business (AOR = 9.79, 95% CI: 4.27–22.43, p < 0.001) and those employed as workers or employees (AOR = 6.38, 95% CI: 2.90–14.04, p < 0.001) had substantially higher odds of undernutrition compared to daily laborers.

Household size was associated with undernutrition; women from households with more than seven members had significantly higher odds (AOR = 1.96, 95% CI: 1.34–2.88, p = 0.001) compared to those in households with fewer than seven members. Urban residence was significantly associated with increased odds of undernutrition. Women residing in urban areas had significantly higher odds of undernutrition (AOR = 2.40, 95% CI: 1.39–4.15, p = 0.002) compared to those residing in rural areas. Income was a significant determinant, with women from households earning less than USD 500 monthly having approximately twice the odds of undernutrition (AOR = 1.98; 95% CI: 1.33–2.94; p = 0.001) compared to those with higher income levels. Lack of access to latrine facilities also increased the likelihood of undernutrition (AOR = 2.00, 95% CI: 1.23–3.26, p = 0.005).

Gestational age was a significant determinant; women in the second trimester (AOR = 8.59, 95% CI: 5.29–13.97; p < 0.001) and third trimester (AOR = 6.91, 95% CI: 2.97–16.08; p < 0.001) were associated with substantially higher odds of undernutrition compared to those in the first trimester. Primigravid women had significantly higher odds of undernutrition compared to multigravida women (AOR = 2.29, 95% CI: 1.52–3.45, p < 0.001). Women with a history of previous contraceptive use were associated with significantly higher odds of undernutrition compared to those with no history of use (AOR = 1.98, 95% CI: 1.34–2.90, p = 0.001). Substance use was strongly associated with undernutrition, with women reporting substance consumption demonstrating nearly threefold increased odds (AOR = 2.79; 95% CI: 1.93–4.04; p < 0.001) compared to non-users. Household food insecurity was independently associated with undernutrition. Pregnant women from severely food-insecure households had significantly higher odds of undernutrition (AOR = 1.76, 95% CI: 1.01–3.08, p = 0.047) compared to those from food-secure households. Furthermore, some associations with notably large effect sizes, particularly those related to gestational age and occupational categories, should be interpreted with caution, as they may reflect residual confounding, small subgroup sizes, or potential model instability, thereby affecting the robustness of these estimates.

## Discussion

The multivariable logistic regression model demonstrated moderate explanatory power, with a Nagelkerke R² value of approximately 0.42. This indicates that approximately 58% of the variability in maternal undernutrition remains unexplained, highlighting the potential influence of unmeasured factors. These may include dietary quantity and caloric intake, micronutrient deficiencies, intra-household food distribution, cultural feeding practices, and displacement status. The absence of these variables may limit the explanatory power of the model and should be considered when interpreting the findings. Although this demonstrates a substantial contribution of the measured factors, a large proportion of the variance remains unexplained. This may be attributable to unmeasured contextual exposures, including protracted conflict, recurrent humanitarian crises, and population displacement. Mogadishu hosts a high concentration of internally displaced persons (IDPs), who are known to experience significantly higher levels of acute malnutrition compared to stable urban residents. The absence of displacement status in the present analysis may partially explain the exceptionally high prevalence of undernutrition observed, representing an important area for future research.

This study found that the prevalence of undernutrition among pregnant women was 75.7%, which is substantially higher than national estimates reported in the Somali Health and Demographic Survey (15%) [20]. This proportion is markedly high, suggesting that maternal undernutrition is a major public health concern in this setting. This discrepancy can be attributed to several important methodological and contextual differences. First, the present study used mid-upper arm circumference (MUAC <23 cm), which is more sensitive to acute undernutrition, whereas national surveys typically rely on body mass index (BMI), which reflects chronic nutritional status. Second, the facility-based design of this study may have captured a higher-risk population, as pregnant women attending ANC services in urban settings are more likely to experience underlying health and nutritional vulnerabilities. Third, Mogadishu is characterized by high levels of urban poverty, population displacement, food insecurity, and reliance on market-based food systems, all of which may contribute to reduced dietary quality and increased risk of undernutrition. These combined factors likely explain the substantially higher prevalence observed in this study. The exceptionally high prevalence observed may also partly reflect the use of a sensitive MUAC cutoff in a predominantly vulnerable urban population attending public referral hospitals.

The prevalence observed in this study is higher than that of studies conducted in the Somali Regional State in Ethiopia (21%) [1], Southwest Ethiopia (42.2%) [29], Kacha Birra District in Southern Ethiopia (52.6%) [30], North Shewa in Ethiopia (22.2%) [31], Tigray in Northern Ethiopia (40.6%) [32], Haramaya District in Eastern Ethiopia (47.9%) [33], Liban District in Ethiopia (44.9%) [34], Chiro District in Eastern Ethiopia (40.9%) [35], Arsi Zone in Ethiopia (48.6%) [36], Konso District in Southern Ethiopia (43.1%) [37], Afar Regional State in Northeast Ethiopia (30.9%) [38], Kenya (27%) [39], Bangladesh (32%) [40], Burundi (34.3%) [41], Tanzania (11%) [42], Zambia (21.8%) [43], Khartoum in Sudan (12.5%) [44], Egypt (31.8%) [45], and systematic review and meta-analysis in the Sub-Saharan Africa region (23.5%) [46], all of which are considerably lower than the findings of our study. This high burden observed in this study could be attributed to several interconnected factors, including prolonged conflict, poverty, recurrent droughts, food insecurity, limited access to quality healthcare and nutritional services, cultural practices influencing diet and health, inadequate awareness about maternal nutrition, and high rates of infectious diseases, all of which may exacerbate nutritional deficiencies and undermine household dietary diversity and access to adequate nutrition.

Age was significantly associated with undernutrition, with women aged 25–38 years being at higher risk compared to those aged 39 years and above. This finding contrasts with studies conducted in Uganda [47]. The increased nutritional risk in these age groups may be attributed to heightened physiological demands and caregiving responsibilities, compounded by socioeconomic constraints. Educational attainment also demonstrated a paradoxical association, whereby women with a tertiary education were more likely to be undernourished than those with no formal education. This finding contrasts with existing evidence in Ethiopia [48], where higher educational status is typically associated with improved nutritional outcomes. However, this result should be interpreted with caution. It may be influenced by residual confounding, small subgroup sizes, or measurement limitations. Additionally, unmeasured socioeconomic factors, such as employment instability or income variability, may partially explain this association. This finding may also reflect the relatively small number of participants with tertiary education in the study population. Further research is needed to better understand this relationship in similar contexts.

Maternal occupation emerged as an important determinant. Pregnant women employed in private business and those employed as employees were significantly more likely to be undernourished compared to daily laborers. Similar findings have been reported in India [49]. The observed occupational differences may reflect variations in socioeconomic conditions, workload patterns, income instability, dietary practices, and access to adequate nutrition among employed women in this setting. However, these findings should be interpreted cautiously because occupational subgroup sizes varied considerably, and residual confounding may persist. Housewives, on the other hand, may have greater opportunities to prioritize rest and food consumption, especially in contexts where men are the primary income earners. Household size was strongly associated with maternal nutritional status. Women living in households with seven or more members were nearly twice as likely to be undernourished compared to those in smaller families. This reflects the principle of “resource dilution,” whereby larger households distribute limited resources across many members, thereby constraining the quantity and quality of food available for pregnant women. Comparable findings have been reported in East Africa [50], where household crowding was associated with poor maternal dietary diversity and undernutrition.

Income levels were significantly associated with nutritional status; women from households earning less than USD 500 monthly had nearly twice the odds of undernutrition compared to those with higher income levels. This finding is corroborated by a study in the Somali Region of Ethiopia [1]where low household income was a determinant of undernutrition among pregnant women. The finding that women living in urban areas had greater odds of undernutrition compared to their rural counterparts contrasts with evidence from a systematic review and meta-analysis in Ethiopia, where rural residence was more commonly associated with maternal malnutrition [4]. This urban disadvantage in Mogadishu could be linked to poverty, overcrowding, displacement, and reliance on purchased food items of poor nutritional quality rather than subsistence farming. Similarly, lack of access to latrine facilities was significantly associated with maternal undernutrition, highlighting the role of sanitation and hygiene in nutritional outcomes. This association aligns with studies in northwest Ethiopia [51], where poor WASH (water, sanitation, and hygiene) practices were linked to increased maternal morbidity and undernutrition.

Gestational age was strongly associated with maternal undernutrition, with women in the second and third trimesters exhibiting significantly higher odds compared to those in the first trimester. Similar associations have been documented in Eastern regions [52], suggesting that as pregnancy advances, maternal nutrient demands increase, and inadequate intake may result in worsening nutritional status. While this finding may reflect increased nutritional demands as pregnancy progresses, it should be interpreted with caution. Physiological changes during pregnancy, including alterations in body composition and fat distribution, may influence mid-upper arm circumference (MUAC) measurements across different gestational stages. As such, MUAC may not remain entirely stable throughout pregnancy, potentially contributing to the observed association. In addition, residual confounding and unmeasured factors may have influenced this relationship. Further investigation is warranted to better understand the dynamics between gestational age and MUAC-based assessment of maternal nutritional status.

Moreover, primigravid women were more likely to be undernourished than multigravida women, corroborating findings from Ethiopia [31], where first-time mothers often lack experience in nutrition and pregnancy care. Previous contraceptive use was positively associated with undernutrition, a finding that may reflect underlying socioeconomic and reproductive health vulnerabilities rather than a direct biological effect. Women with a history of contraceptive use may represent those with closely spaced pregnancies, prior nutritional depletion, or limited access to comprehensive reproductive health and nutrition services. Similar associations have been reported in other low-resource settings, where contraceptive use serves as a proxy for broader reproductive health challenges rather than a protective factor in isolation. Substance use was also strongly associated with undernutrition, consistent with reports from Ethiopia [53], where khat chewing and alcohol consumption were linked to poor maternal nutritional outcomes. Furthermore, household food insecurity was significantly associated with maternal undernutrition. Women from severely food-insecure households had higher odds of undernutrition compared to those from food-secure households. This finding is consistent with evidence from a study in the Somali region of Ethiopia [1], indicating that food insecurity is an important contributing factor to maternal undernutrition, as it directly limits access to adequate and nutritious food and contributes to poor dietary quality. Furthermore, the high prevalence of severe household food insecurity observed in this study suggests a potential interaction with other socioeconomic and demographic factors, including income level, household size, and maternal occupation, in shaping maternal nutritional outcomes. However, these interactions were not formally examined in the present analysis and should be explored in future research to better understand their combined effects.

### Strengths and limitations

This study is among the first to document the prevalence and determinants of maternal undernutrition in Mogadishu, using a large sample size, a high response rate, and standardized tools such as MUAC and HFIAS, which strengthen the validity of the findings. The use of multivariable analysis also allowed for robust identification of independent predictors. However, this study has several limitations that should be considered when interpreting the findings. First, the cross-sectional design limits causal inference, and reliance on self-reported data may have introduced recall bias. Second, the facility-based design may have introduced selection bias, as only pregnant women attending antenatal care services were included, thereby excluding those who do not access health facilities and potentially limiting the generalizability of the results. Third, dietary intake was assessed using a 24-hour recall method, which is subject to recall bias and may not accurately reflect habitual dietary patterns. Additionally, although multiple variables were included in the analysis, residual confounding may persist due to unmeasured factors such as displacement status, cultural dietary practices, and intra-household food allocation. The disproportionately high proportion of first-trimester participants may reflect antenatal care attendance patterns, referral dynamics, or sampling variation during the study period and may have limited the representativeness of gestational age-related findings. These limitations should be taken into account when interpreting the study findings.

## Conclusion

This study demonstrated a high prevalence of maternal undernutrition among pregnant women attending antenatal care services in public hospitals in Mogadishu. Maternal age, educational level, occupation, household size, residence, income, sanitation access, gestational age, gravidity, contraceptive use, substance use, and household food insecurity were identified as being associated with maternal undernutrition. These findings highlight the need for targeted, multi-sectoral interventions aimed at improving maternal nutritional status, including strengthening food security, enhancing nutrition education, and improving access to quality maternal healthcare services. However, these recommendations are informed by observed associations and should be interpreted with caution, as causal relationships cannot be established from this study. Integrating maternal nutrition screening and targeted nutritional support into routine antenatal care services is essential in fragile and humanitarian settings such as Somalia.

## Abbreviations

AOR: adjusted odds ratio
ANC: antenatal care
BMI: body mass index
CI: confidence interval
COR: crude odds ratio
FMOH: Federal Ministry of Health
HFIAS: household food insecurity access scale
IRB: institutional review board
IUGR: intrauterine growth restriction
LMICs: low- and middle-income countries
MUAC: mid-upper arm circumference
RIC: research and innovation center
SDGs: sustainable development goals
SPSS: statistical package for the social sciences
UNICEF: United Nations Children’s Fund
USD: United States Dollar
WASH: water, sanitation, and hygiene
WHO: World Health Organization
ZUST: Zamzam University of Science and Technology
