# Supplementary material for: Prevalence of undernutrition and its associated factors among pregnant women attending antenatal care service in public hospitals in Mogadishu, Somalia
Source: PLoS One. 2026 Jul 7;21(7):e0347187. doi: 10.1371/journal.pone.0347187 (PMC13340802; doi:10.1371/journal.pone.0347187)
Supplement: Attachment — Submitted filename: Point-by-point Reviewers Response.docx [file pone.0347187.s001.docx]

**POINT-BY-POINT REVIEWER'S RESPONSE**

| **Reviewer’s Comment** | **Author’s Response** |
| --- | --- |
| **Preamble:** This study assessed the prevalence and associated factors of undernutrition among pregnant women attending antenatal care (ANC) services in public hospitals in Mogadishu. The findings may inform public health interventions and help in designing strategies to enhance maternal nutrition. | We thank the reviewer for this positive overview of the study and for recognizing its potential contribution to informing public health interventions and maternal nutrition strategies in Mogadishu, Somalia. |
| **INTRODUCTION**: There is a general problem of referencing sentences because the citations are placed after “a full stop”. For example, “Undernutrition among pregnant women remains a significant public health challenge worldwide, particularly in low- and middle-income countries such as Somalia, where socioeconomic disparities, limited healthcare access, and food insecurity are prevalent. [1]” | We appreciate the reviewer’s observation. The manuscript has been carefully revised to ensure that all citations are placed according to standard academic referencing conventions. Specifically, references are now positioned before the final punctuation in accordance with Vancouver citation style (e.g., “…food insecurity are prevalent [1].”). This correction has been applied consistently throughout the entire manuscript, particularly in the Introduction section. |
| To address the problem of citation placement, the authors should follow standard academic formatting rules based on the specific style guide. | The authors have fully adhered to this recommendation. All in-text citations have been reformatted to comply with the journal’s referencing style, ensuring consistency and adherence to accepted academic standards. |
| The justification for the study appears unclear… What form of undernutrition are the authors seeking to investigate in the present study? The authors should clearly explain what is so unique about the study and how their assessment of undernutrition of pregnant women is different from what is reported in the Somali Health and Demographic Survey 2020 and other similar studies carried out in the country. | We thank the reviewer for highlighting this important point. The Introduction has been substantially revised to clearly clarify the study’s justification and uniqueness. We now explicitly state that while the Somali Health and Demographic Survey (SHDS) 2020 reports national estimates of maternal undernutrition primarily based on BMI, the present study focuses on acute maternal undernutrition assessed using mid-upper arm circumference (MUAC < 23 cm). MUAC is a sensitive indicator recommended for identifying current nutritional risk among pregnant women, particularly in emergency and resource-limited settings such as Mogadishu. Furthermore, this study is facility-based, urban-specific, and incorporates contextual determinants such as household food insecurity, occupation, and sanitation, which are not adequately captured in national surveys. |
| **METHODS –** Assessment of dietary intake and food security: Household Food Insecurity Access Scale (HFIAS): the authors should provide details of how this was actually measured. What were the parameters used? | The Methods section has been revised to include a detailed description of the HFIAS. We now specify that the scale consists of nine occurrence questions assessing anxiety about food supply, food quality, and food quantity over the previous four weeks, followed by frequency-of-occurrence questions. Households were categorized into food secure, mildly, moderately, and severely food insecure according to standard HFIAS guidelines. |
| Explain how dietary intake patterns of pregnant women were classified as either adequate or inadequate. | We have clarified this in the Methods section. Dietary intake was assessed using a 24-hour dietary recall, and consumption was categorized as “adequate” if at least one food item from a given food group was consumed during the recall period and “inadequate” if none was consumed. This approach is consistent with commonly used dietary diversity assessment methods in similar settings. |
| **Statistical analysis:** Before applying binary logistic regression, how was the presence of multicollinearity among the independent variables diagnosed? | We appreciate this comment. The Data Analysis subsection has been updated to indicate that multicollinearity was assessed using Variance Inflation Factors (VIF). A VIF value greater than 10 was considered indicative of multicollinearity, and no such values were observed, confirming the suitability of the regression model. |
| **RESULTS:** The finding that attainment of tertiary education was a risk factor for undernutrition is unusual… | We acknowledge the reviewer’s concern. The Results section now includes a brief clarifying statement noting that this finding likely reflects contextual socioeconomic realities rather than an inherent risk of education itself. No changes were made to the statistical results, but interpretation has been carefully contextualized. |
| **DISCUSSION:** As quantified by the Nagelkerke R Square in the logistic regression analyses, what percentage of the variance of maternal undernutrition is accounted for by the measured risk factors? | Thank you for this important observation. The Discussion section has been revised to report the Nagelkerke R² value, indicating the proportion of variance in maternal undernutrition explained by the model. We further acknowledge that a substantial proportion of the variance remains unexplained, suggesting the influence of unmeasured factors. |
| The 75% of undernutrition in Mogadishu is really alarming and this may be attributed to an unmeasured exposure including the ongoing humanitarian crisis and displacement… | We fully agree with the reviewer. The Discussion has been expanded to highlight the role of prolonged humanitarian crises, urban poverty, and the high concentration of internally displaced persons (IDPs) in Mogadishu as plausible contributors to the extremely high prevalence of maternal undernutrition observed in this study. We also acknowledge displacement status as an important unmeasured variable and recommend its inclusion in future research. |
| What possibly explains why attainment of tertiary education was a risk factor for undernutrition among pregnant women? | The Discussion has been strengthened to provide a detailed contextual explanation. We now discuss how tertiary education in Somalia does not necessarily translate into stable employment or improved income for women, particularly in Mogadishu, where unemployment, informal labor, and occupational stress may override the expected nutritional benefits of education. |
| Similarly, previous contraceptive use associated positively with undernutrition. What accounts for this? And are there similar results in the literature? | We thank the reviewer for this insightful comment. A new paragraph has been added to the Discussion explaining that previous contraceptive use may reflect cumulative reproductive stress, short birth intervals, or underlying socioeconomic vulnerability rather than a direct causal effect. We also note that similar associations have been reported in other low-resource settings and cite supporting literature accordingly. |
