# Supplementary material for: Prevalence of undernutrition and its associated factors among pregnant women attending antenatal care service in public hospitals in Mogadishu, Somalia
Source: PLoS One. 2026 Jul 7;21(7):e0347187. doi: 10.1371/journal.pone.0347187 (PMC13340802; doi:10.1371/journal.pone.0347187)
Supplement: Attachment — Submitted filename: Response to Reviewers.docx [file pone.0347187.s002.docx]

**POINT-BY-POINT REVIEWER'S RESPONSE**

| **Reviewer 1** | |
| --- | --- |
| **Reviewer’s Comment** | **Author’s Response** |
| **METHODS – Assessment of dietary diversity, and food security** |  |
| i. Household Food Insecurity Access Scale (HFIAS): the authors should provide the reference that gives details of how this was actually measured? | Thank you for this important comment. We have revised the Methods section to clearly describe the HFIAS measurement and included the appropriate reference (FANTA guideline). Specifically, we now state that HFIAS consists of nine standardized occurrence questions covering three domains (anxiety, food quality, and food intake), with frequency-of-occurrence responses used to classify households into four categories (food secure, mildly, moderately, and severely food insecure). |
| ii. The explanation given for classifying dietary intake patterns of pregnant women as either adequate or inadequate is still not clear. According to the authors, food items consumed were grouped according to standard food groups (what does “standard” refer to here? how many food groups were involved). | We appreciate this clarification request. The Methods section has been revised to explicitly define “standard food groups” as the ten food groups specified by the Minimum Dietary Diversity for Women (MDD-W) indicator. These ten groups are now clearly listed in the manuscript to ensure transparency and reproducibility. |
| The authors also categorized dietary intake as “adequate” if at least one food item from a given food group was consumed during the recall period and “inadequate” if none was consumed. | Thank you for highlighting this ambiguity. We have revised the manuscript to clarify that consumption of individual food groups was used only to describe dietary patterns, not to determine overall dietary adequacy. |
| So, if a woman was classified as adequate for a particular food group but inadequate for another food group, how then did the authors describe the dietary intake patterns of this woman? | We have addressed this concern by clearly distinguishing between food group consumption and overall dietary adequacy. Specifically, we now state that overall dietary adequacy was determined using the Dietary Diversity Score (DDS), which summarizes the number of food groups consumed. Individual food group consumption is presented descriptively and does not determine overall adequacy. |
| Let me just mention that there is a difference between food variety and dietary diversity. | We thank the reviewer for this important distinction. The Methods section has been revised to explicitly differentiate between food variety (number of individual food items consumed) and dietary diversity (number of food groups consumed). This clarification aligns with established nutritional assessment frameworks. |
| Food Variety Score (FVS): This is a simple count of the individual food items consumed over a specific period… | We acknowledge this explanation and have incorporated the conceptual distinction into the manuscript. We clarify that our study used Dietary Diversity Score (DDS), not Food Variety Score (FVS), to assess overall diet quality. |
| Dietary Diversity Score (DDS): This measures the number of food groups represented in a diet… | We have revised the Methods section to clearly describe DDS calculation based on the ten MDD-W food groups. We also specified the cut-off point, where consumption of ≥5 food groups indicate adequate dietary diversity, consistent with international guidelines. |
| **STUDY RESULTS** |  |
| The results presented in Table 4 could be improved to show the overall dietary diversity of pregnant women. | Thank you for this suggestion. We have revised the Results section to prioritize Dietary Diversity Score (DDS) as the main indicator of overall dietary adequacy. The DDS findings are now presented first before describing individual food group consumption. |
| Dietary adequacy should not be based only on individual food groups but all the food groups. | We agree and have addressed this by clarifying that overall dietary adequacy is determined using DDS (based on all food groups combined), rather than individual food groups. Individual food group consumption is now clearly presented as descriptive information only. |
| For example, the minimum dietary diversity for women (MDD-W) is achieved if the woman consumes five or more of the 10 defined food groups. | This recommendation has been incorporated into the manuscript. We now explicitly state that adequate dietary diversity is defined as consumption of at least five out of the ten MDD-W food groups. |
| Studies indicate that dietary diversity is often a more effective predictor of overall nutrient adequacy… | We appreciate this insight and have strengthened the manuscript by emphasizing that DDS is a proxy indicator of micronutrient adequacy and overall diet quality, consistent with established literature. Additionally, the Results section has been revised to reflect this emphasis. |

| **Reviewer 2** | |
| --- | --- |
| **Reviewer’s Comment** | **Author’s Response** |
| 1. The reported prevalence of undernutrition (75.7%) is exceptionally high and requires contextual qualification. Please briefly acknowledge in the Abstract that this estimate is based on MUAC (<23 cm) and a facility-based sample, which may limit comparability with national estimates. | We thank the reviewer for this important observation. The Abstract has been revised to clearly indicate that the reported prevalence is based on MUAC (<23 cm) and a facility-based sample, which may limit comparability with national estimates derived from BMI-based population surveys. |
| 2. (Results section): Several adjusted odds ratios (e.g., private business AOR = 9.79; second trimester AOR = 8.59) appear unusually large. Consider adding a short cautionary phrase indicating that these associations should be interpreted carefully. | We appreciate this comment. A cautionary statement has been added to the Results section indicating that large effect sizes should be interpreted with caution due to potential residual confounding, small subgroup sizes, or model instability. |
| 3. While the background is comprehensive, the distinction between BMI-based national estimates and MUAC-based assessment should be more clearly and concisely articulated earlier in the section to strengthen the study rationale. | We thank the reviewer for this suggestion. The Introduction has been revised to more clearly and concisely distinguish between BMI-based national estimates and MUAC-based assessment earlier in the section. |
| 4. The justification for focusing on an urban, facility-based population is improved but could be further sharpened by explicitly stating why ANC attendees in Mogadishu represent a high-risk subgroup. | We appreciate this comment. The Introduction has been strengthened by explicitly stating that ANC attendees in Mogadishu represent a high-risk subgroup due to urban poverty, displacement, and food insecurity. |
| Methods (Study Design): The facility-based cross-sectional design should be more explicitly acknowledged as limiting generalizability, particularly given that women not attending ANC are excluded. | We thank the reviewer for this important point. The limitation of the facility-based cross-sectional design in restricting generalizability has been explicitly acknowledged in the Discussion section. |
| 5. (Sampling): Additional clarity is needed regarding how the sampling interval (k) was operationalized in each hospital. Providing a brief example would improve reproducibility. | We appreciate this suggestion. A detailed example illustrating how the sampling interval (k) was calculated and applied has been added to improve clarity and reproducibility. |
| 6. (Anthropometry): The use of MUAC <23 cm as a cutoff requires stronger justification with citation of WHO or emergency nutrition guidelines, as this threshold critically influences the prevalence estimate. | We thank the reviewer. The manuscript has been revised to include stronger justification for the MUAC <23 cm cutoff, supported by WHO and emergency nutrition guidelines. |
| 7. (Dietary Assessment): The classification of dietary intake as “adequate” based on consumption of at least one item per food group over 24 hours appears overly simplistic. This should be reframed as “food group consumption” rather than adequacy. | We appreciate this important comment. The terminology has been revised throughout the manuscript to reflect “food group consumption (Yes/No)” rather than adequacy, and overall dietary adequacy is now determined using DDS. |
| 8. (Statistical Analysis): While VIF was used to assess multicollinearity, additional details on model-building strategy (e.g., theoretical vs stepwise selection) would strengthen transparency. The choice of p<0.25 for inclusion in multivariable analysis should be justified with a citation or brief rationale. | We thank the reviewer. The model-building strategy has been clarified to include both statistical and theoretical considerations, and justification for the use of p<0.25 has been provided. |
| 9. (Prevalence): The very high prevalence (75.7%) requires further descriptive support. Reporting the mean and distribution (e.g., SD or percentiles) of MUAC would help readers assess plausibility. | We appreciate this comment. The mean and standard deviation of MUAC have been included to provide additional descriptive support for the reported prevalence. |
| 10. (Table 5): Some categories (e.g., tertiary education, n=53) are relatively small, yet show strong associations (AOR = 3.43). Please comment on the stability of these estimates. | We thank the reviewer. A statement has been added acknowledging that small subgroup sizes may affect the stability and precision of the estimates. |
| 11. (Regression findings): The magnitude of certain associations (e.g., second trimester AOR = 8.59; private business AOR = 9.79) raises concern for residual confounding or model instability. This should be acknowledged. | We appreciate this comment. A statement has been added acknowledging that large effect sizes may reflect residual confounding or model instability. |
| 12. Interpretive language is occasionally used (e.g., explanation of education findings). Interpretation should be reserved for the Discussion section. | We thank the reviewer. Interpretive language has been removed from the Results section and appropriately relocated to the Discussion section. |
| 13. (Prevalence): While comparisons with other countries are extensive, the discussion should more critically address why the prevalence in this study is substantially higher than both national (15%) and regional estimates. | We appreciate this comment. The Discussion has been strengthened to critically explain the higher prevalence based on methodological and contextual differences. |
| 14. (Education finding): The explanation provided for tertiary education being associated with undernutrition is speculative. It would be helpful to explicitly acknowledge alternative explanations such as confounding or measurement bias. | We thank the reviewer. The explanation has been revised to reduce speculation and to acknowledge potential confounding and measurement bias. |
| 15. (Trimester effect): The very strong association between trimester and undernutrition requires cautious interpretation. Consider discussing whether MUAC changes across pregnancy stages may influence this finding. | We appreciate this suggestion. The Discussion now includes a cautious interpretation and consideration of physiological changes affecting MUAC during pregnancy. |
| 16. (Model performance): The reported Nagelkerke R² (~42%) is mentioned but not critically interpreted. Please clarify what proportion of variability remains unexplained and what key variables may be missing. | We thank the reviewer. The Discussion has been expanded to interpret the unexplained variance and identify potential missing variables. |
| 17. (Bias): Selection bias (facility-based sample), recall bias (24-hour dietary recall), and unmeasured confounders (e.g., displacement status) should be more explicitly discussed. | We appreciate this comment. The limitations section has been strengthened to explicitly address these biases. |
| 18. (Food insecurity): Given that over half of households were severely food insecure, the interaction between food insecurity and other predictors could be explored or at least acknowledged. | We thank the reviewer. A statement has been added acknowledging potential interactions and recommending further research. |
| 19. The conclusions are currently somewhat strong relative to the observational design. Statements implying causality should be softened (e.g., replace “determinants” with “associated factors”). | We appreciate this comment. The Conclusion has been revised to remove causal language and replace it with associative terminology. |
| 20. Policy recommendations are appropriate but should be framed as being based on associative evidence rather than causal inference. | We thank the reviewer. Policy recommendations have been reframed to reflect that they are based on observed associations. |
| 21. Data Availability: The statement that data are “available upon reasonable request” does not meet PLOS ONE requirements. Please revise to include a public repository or provide a justified exception. | We appreciate this comment. The data availability statement has been revised to reflect ethical restrictions and IRB approval requirements. |
| 22. Overall clarity: The manuscript is generally well-structured, but minor language refinement would improve readability, particularly in the Results and Discussion sections. | We thank the reviewer for this helpful suggestion. The manuscript has been carefully revised to improve clarity, coherence, and readability, particularly in the Results and Discussion sections. |
