# Supplementary material for: Prevalence of undernutrition and its associated factors among pregnant women attending antenatal care service in public hospitals in Mogadishu, Somalia
Source: PLoS One. 2026 Jul 7;21(7):e0347187. doi: 10.1371/journal.pone.0347187 (PMC13340802; doi:10.1371/journal.pone.0347187)
Supplement: Peer review history

# Peer review: Decision Letter 0

**DOI:** 10.1371/journal.pone.0347187.r001

**Authors:** Jianhong Zhou

8 Jan 2026

PONE-D-25-46984

Prevalence of Undernutrition and Its Associated Factors Among Pregnant Women Attending Antenatal Care Service in Public Hospitals in Mogadishu, Somalia

PLOS One

Dear Dr. Tahlil,

Thank you for submitting your manuscript to PLOS ONE. After careful consideration, we feel that it has merit but does not fully meet PLOS ONE’s publication criteria as it currently stands. Therefore, we invite you to submit a revised version of the manuscript that addresses the points raised during the review process.

Please submit your revised manuscript by Feb 21 2026 11:59PM. If you will need more time than this to complete your revisions, please reply to this message or contact the journal office at plosone@plos.org. When you're ready to submit your revision, log on to https://www.editorialmanager.com/pone/ and select the 'Submissions Needing Revision' folder to locate your manuscript file.

Please include the following items when submitting your revised manuscript:

- A letter that responds to each point raised by the academic editor and reviewer(s). You should upload this letter as a separate file labeled 'Response to Reviewers'.
- A marked-up copy of your manuscript that highlights changes made to the original version. You should upload this as a separate file labeled 'Revised Manuscript with Track Changes'.
- An unmarked version of your revised paper without tracked changes. You should upload this as a separate file labeled 'Manuscript'.

If you would like to make changes to your financial disclosure, please include your updated statement in your cover letter. Guidelines for resubmitting your figure files are available below the reviewer comments at the end of this letter.

If applicable, we recommend that you deposit your laboratory protocols in protocols.io to enhance the reproducibility of your results. Protocols.io assigns your protocol its own identifier (DOI) so that it can be cited independently in the future. For instructions see: https://journals.plos.org/plosone/s/submission-guidelines#loc-laboratory-protocols. Additionally, PLOS ONE offers an option for publishing peer-reviewed Lab Protocol articles, which describe protocols hosted on protocols.io. Read more information on sharing protocols at https://plos.org/protocols?utm_medium=editorial-email&utm_source=authorletters&utm_campaign=protocols.

We look forward to receiving your revised manuscript.

Kind regards,

Jianhong Zhou

Staff Editor

PLOS One

Journal Requirements:

When submitting your revision, we need you to address these additional requirements.

1. Please ensure that your manuscript meets PLOS ONE's style requirements, including those for file naming. The PLOS ONE style templates can be found at

https://journals.plos.org/plosone/s/file?id=wjVg/PLOSOne_formatting_sample_main_body.pdf and

https://journals.plos.org/plosone/s/file?id=ba62/PLOSOne_formatting_sample_title_authors_affiliations.pdf

2. We note that you have indicated that there are restrictions to data sharing for this study. For studies involving human research participant data or other sensitive data, we encourage authors to share de-identified or anonymized data. However, when data cannot be publicly shared for ethical reasons, we allow authors to make their data sets available upon request. For information on unacceptable data access restrictions, please see http://journals.plos.org/plosone/s/data-availability#loc-unacceptable-data-access-restrictions.

Before we proceed with your manuscript, please address the following prompts:

a) If there are ethical or legal restrictions on sharing a de-identified data set, please explain them in detail (e.g., data contain potentially identifying or sensitive patient information, data are owned by a third-party organization, etc.) and who has imposed them (e.g., a Research Ethics Committee or Institutional Review Board, etc.). Please also provide contact information for a data access committee, ethics committee, or other institutional body to which data requests may be sent.

b) If there are no restrictions, please upload the minimal anonymized data set necessary to replicate your study findings to a stable, public repository and provide us with the relevant URLs, DOIs, or accession numbers. Please see http://www.bmj.com/content/340/bmj.c181.long for guidelines on how to de-identify and prepare clinical data for publication. For a list of recommended repositories, please see https://journals.plos.org/plosone/s/recommended-repositories. You also have the option of uploading the data as Supporting Information files, but we would recommend depositing data directly to a data repository if possible.

Please update your Data Availability statement in the submission form accordingly.

3. In this instance it seems there may be acceptable restrictions in place that prevent the public sharing of your minimal data. However, in line with our goal of ensuring long-term data availability to all interested researchers, PLOS’ Data Policy states that authors cannot be the sole named individuals responsible for ensuring data access (http://journals.plos.org/plosone/s/data-availability#loc-acceptable-data-sharing-methods).

Data requests to a non-author institutional point of contact, such as a data access or ethics committee, helps guarantee long term stability and availability of data. Providing interested researchers with a durable point of contact ensures data will be accessible even if an author changes email addresses, institutions, or becomes unavailable to answer requests.

Before we proceed with your manuscript, please also provide non-author contact information (phone/email/hyperlink) for a data access committee, ethics committee, or other institutional body to which data requests may be sent. If no institutional body is available to respond to requests for your minimal data, please consider if there any institutional representatives who did not collaborate in the study, and are not listed as authors on the manuscript, who would be able to hold the data and respond to external requests for data access? If so, please provide their contact information (i.e., email address). Please also provide details on how you will ensure persistent or long-term data storage and availability.

4. PLOS requires an ORCID iD for the corresponding author in Editorial Manager on papers submitted after December 6th, 2016. Please ensure that you have an ORCID iD and that it is validated in Editorial Manager. To do this, go to ‘Update my Information’ (in the upper left-hand corner of the main menu), and click on the Fetch/Validate link next to the ORCID field. This will take you to the ORCID site and allow you to create a new iD or authenticate a pre-existing iD in Editorial Manager.

5. Your ethics statement should only appear in the Methods section of your manuscript. If your ethics statement is written in any section besides the Methods, please move it to the Methods section and delete it from any other section. Please ensure that your ethics statement is included in your manuscript, as the ethics statement entered into the online submission form will not be published alongside your manuscript.

6. If the reviewer comments include a recommendation to cite specific previously published works, please review and evaluate these publications to determine whether they are relevant and should be cited. There is no requirement to cite these works unless the editor has indicated otherwise.

**Additional Editor Comments:**

Please note that we have only been able to secure a single reviewer to assess your manuscript. We are issuing a decision on your manuscript at this point to prevent further delays in the evaluation of your manuscript. Please be aware that the editor who handles your revised manuscript might find it necessary to invite additional reviewers to assess this work once the revised manuscript is submitted. However, we will aim to proceed on the basis of this single review if possible.

[Note: HTML markup is below. Please do not edit.]

Reviewers' comments:

Reviewer's Responses to Questions

**Comments to the Author**

1. Is the manuscript technically sound, and do the data support the conclusions?

The manuscript must describe a technically sound piece of scientific research with data that supports the conclusions. Experiments must have been conducted rigorously, with appropriate controls, replication, and sample sizes. The conclusions must be drawn appropriately based on the data presented.

Reviewer #1: Partly

**********

2. Has the statistical analysis been performed appropriately and rigorously?

Reviewer #1: Yes

**********

3. Have the authors made all data underlying the findings in their manuscript fully available?

The PLOS Data policy requires authors to make all data underlying the findings described in their manuscript fully available without restriction, with rare exception (please refer to the Data Availability Statement in the manuscript PDF file). The data should be provided as part of the manuscript or its supporting information, or deposited to a public repository. For example, in addition to summary statistics, the data points behind means, medians and variance measures should be available. If there are restrictions on publicly sharing data—e.g. participant privacy or use of data from a third party—those must be specified.

Reviewer #1: Yes

**********

4. Is the manuscript presented in an intelligible fashion and written in standard English?

PLOS ONE does not copyedit accepted manuscripts, so the language in submitted articles must be clear, correct, and unambiguous. Any typographical or grammatical errors should be corrected at revision, so please note any specific errors here.

Reviewer #1: Yes

**********

5. Review Comments to the Author

Please use the space provided to explain your answers to the questions above. You may also include additional comments for the author, including concerns about dual publication, research ethics, or publication ethics. (Please upload your review as an attachment if it exceeds 20,000 characters)

Reviewer #1: Preamble: This study assessed the prevalence and associated factors of undernutrition among

pregnant women attending antenatal care (ANC) services in public hospitals in Mogadishu. The findings may inform public health interventions and help in designing strategies to enhance maternal nutrition.

Main Concerns

The following are some concerns that need to be addressed to make the paper publishable.

INTRODUCTION

• There is a general problem of referencing sentences because the citations are placed after “a full stop”. For example, “Undernutrition among pregnant women remains a significant public health challenge worldwide, particularly in low- and middle-income countries such as Somalia, where socioeconomic disparities, limited healthcare access, and food insecurity are prevalent. [1]”

• To address the problem of citation placement, the authors should follow standard academic formatting rules based on the specific style guide:

Standard Placement (Before the Period)

i. In most citation styles, including AMA, APA, and MLA, the reference is considered part of the sentence and must be placed before the final punctuation.

ii. Example (APA/MLA): ...particularly in low- and middle-income countries such as Somalia (Smith, 2025).

iii. Example (Vancouver/AMA): ...limited healthcare access and food insecurity are prevalent [1].

• The justification for the study appears unclear because the authors have stated on pages 2-3 that “According to the Somali Health and Demographic Survey 2020, approximately 15% of pregnant women in Somalia experience undernutrition. [20]”. However, the authors went ahead to indicate that “Despite these challenges, evidence on the prevalence and determinants of undernutrition among pregnant women in Mogadishu remains limited. Existing studies in Somalia have primarily focused on anemia or child malnutrition. This study aims to assess the prevalence of undernutrition and its associated factors among pregnant women attending ANC services in public hospitals in Mogadishu, Somalia’ What form of undernutrition are the authors seeking to investigate in the present study? The authors should clearly explain what is so unique about the study and how their assessment of undernutrition of pregnant women is different from what is reported in the Somali Health and Demographic Survey 2020 and other similar studies carried out in the country.

METHODS

Assessment of dietary intake, and food security

i. Household Food Insecurity Access Scale (HFIAS): the authors should provide details of how this was actually measured. What were the parameters used?

ii. Explain how dietary intake patterns of pregnant women were classified as either adequate or inadequate.

Statistical analysis

Before applying binary logistic regression, how was the presence of multi-collinearity among the independent variable diagnosed?

RESULTS

• The finding that attainment of tertiary education was a risk factor for undernutrition (AOR= 3.43, 95% CI: 1.89–6.21) is unusual, as higher education is typically associated with better nutritional outcomes due to increased knowledge and income potential. Ultimately, the finding is likely a result of local socioeconomic realities and contextual factors that override the typical benefits of education, rather than an inherent risk of education itself.

• This counter-intuitive result is likely explained by specific contextual factors of the study area, which may include:

a. Socioeconomic Disparities: Women with higher education might still face significant economic constraints, such as high unemployment or low income, if the local job market is poor. Undernutrition is often strongly linked to low household income and food insecurity, regardless of education level.

b. Competing Demands and Lifestyle: Pursuing tertiary education or related careers can involve time constraints and high stress, potentially leading to poor dietary practices, meal skipping, or reliance on less nutritious convenience foods.

DISCUSSION OF STUDY RESULTS

i. As quantified by the Nagelkerke R Square in the logistic regression analyses, what percentage of the variance of maternal undernutrition is accounted for by the measured risk factors? The 75 % of undernutrition in Mogadishu is really alarming and this may be attributed to an unmeasured exposure including the ongoing humanitarian crisis and displacement. Mogadishu being the national capital city could be hosting millions displaced persons (IDP Concentrations). These populations often show much higher rates of acute malnutrition than stable urban residents. It would therefore be interesting to see how undernutrition varies with whether respondents were normal residents or displaced.

ii. What possibly explains why attainment of tertiary education was risk factor for undernutrition among pregnant women. This counter-intuitive result is likely explained by specific contextual factors of the study area, which may include:

a. Socioeconomic Disparities: Women with higher education might still face significant economic constraints, such as high unemployment or low income, if the local job market is poor. Undernutrition is often strongly linked to low household income and food insecurity, regardless of education level.

b. Competing Demands and Lifestyle: Pursuing tertiary education or related careers can involve time constraints and high stress, potentially leading to poor dietary practices, meal skipping, or reliance on less nutritious convenience foods.

iii. Similarly, previous contraceptive use associated positively with undernutrition. What accounts for this? And are there similar results in the literature?

**********

6. PLOS authors have the option to publish the peer review history of their article (what does this mean?). If published, this will include your full peer review and any attached files.

If you choose “no”, your identity will remain anonymous but your review may still be made public.

**Do you want your identity to be public for this peer review?**  For information about this choice, including consent withdrawal, please see our Privacy Policy.

Reviewer #1: **Yes:**  Dr. Mahama Saaka

**********

[NOTE: If reviewer comments were submitted as an attachment file, they will be attached to this email and accessible via the submission site. Please log into your account, locate the manuscript record, and check for the action link "View Attachments". If this link does not appear, there are no attachment files.]

To ensure your figures meet our technical requirements, please review our figure guidelines: https://journals.plos.org/plosone/s/figures

You may also use PLOS’s free figure tool, NAAS, to help you prepare publication quality figures: https://journals.plos.org/plosone/s/figures#loc-tools-for-figure-preparation.

NAAS will assess whether your figures meet our technical requirements by comparing each figure against our figure specifications.

# Author comment: Author response to Decision Letter 1

**DOI:** 10.1371/journal.pone.0347187.r002

28 Jan 2026

POINT-BY-POINT REVIEWER'S RESPONSE

Reviewer’s Comment Author’s Response

Preamble: This study assessed the prevalence and associated factors of undernutrition among pregnant women attending antenatal care (ANC) services in public hospitals in Mogadishu. The findings may inform public health interventions and help in designing strategies to enhance maternal nutrition. We thank the reviewer for this positive overview of the study and for recognizing its potential contribution to informing public health interventions and maternal nutrition strategies in Mogadishu, Somalia.

INTRODUCTION: There is a general problem of referencing sentences because the citations are placed after “a full stop”. For example, “Undernutrition among pregnant women remains a significant public health challenge worldwide, particularly in low- and middle-income countries such as Somalia, where socioeconomic disparities, limited healthcare access, and food insecurity are prevalent. [1]” We appreciate the reviewer’s observation. The manuscript has been carefully revised to ensure that all citations are placed according to standard academic referencing conventions. Specifically, references are now positioned before the final punctuation in accordance with Vancouver citation style (e.g., “…food insecurity are prevalent [1].”). This correction has been applied consistently throughout the entire manuscript, particularly in the Introduction section.

To address the problem of citation placement, the authors should follow standard academic formatting rules based on the specific style guide. The authors have fully adhered to this recommendation. All in-text citations have been reformatted to comply with the journal’s referencing style, ensuring consistency and adherence to accepted academic standards.

The justification for the study appears unclear… What form of undernutrition are the authors seeking to investigate in the present study? The authors should clearly explain what is so unique about the study and how their assessment of undernutrition of pregnant women is different from what is reported in the Somali Health and Demographic Survey 2020 and other similar studies carried out in the country. We thank the reviewer for highlighting this important point. The Introduction has been substantially revised to clearly clarify the study’s justification and uniqueness. We now explicitly state that while the Somali Health and Demographic Survey (SHDS) 2020 reports national estimates of maternal undernutrition primarily based on BMI, the present study focuses on acute maternal undernutrition assessed using mid-upper arm circumference (MUAC < 23 cm). MUAC is a sensitive indicator recommended for identifying current nutritional risk among pregnant women, particularly in emergency and resource-limited settings such as Mogadishu. Furthermore, this study is facility-based, urban-specific, and incorporates contextual determinants such as household food insecurity, occupation, and sanitation, which are not adequately captured in national surveys.

METHODS – Assessment of dietary intake and food security: Household Food Insecurity Access Scale (HFIAS): the authors should provide details of how this was actually measured. What were the parameters used? The Methods section has been revised to include a detailed description of the HFIAS. We now specify that the scale consists of nine occurrence questions assessing anxiety about food supply, food quality, and food quantity over the previous four weeks, followed by frequency-of-occurrence questions. Households were categorized into food secure, mildly, moderately, and severely food insecure according to standard HFIAS guidelines.

Explain how dietary intake patterns of pregnant women were classified as either adequate or inadequate. We have clarified this in the Methods section. Dietary intake was assessed using a 24-hour dietary recall, and consumption was categorized as “adequate” if at least one food item from a given food group was consumed during the recall period and “inadequate” if none was consumed. This approach is consistent with commonly used dietary diversity assessment methods in similar settings.

Statistical analysis: Before applying binary logistic regression, how was the presence of multicollinearity among the independent variables diagnosed? We appreciate this comment. The Data Analysis subsection has been updated to indicate that multicollinearity was assessed using Variance Inflation Factors (VIF). A VIF value greater than 10 was considered indicative of multicollinearity, and no such values were observed, confirming the suitability of the regression model.

RESULTS: The finding that attainment of tertiary education was a risk factor for undernutrition is unusual… We acknowledge the reviewer’s concern. The Results section now includes a brief clarifying statement noting that this finding likely reflects contextual socioeconomic realities rather than an inherent risk of education itself. No changes were made to the statistical results, but interpretation has been carefully contextualized.

DISCUSSION: As quantified by the Nagelkerke R Square in the logistic regression analyses, what percentage of the variance of maternal undernutrition is accounted for by the measured risk factors? Thank you for this important observation. The Discussion section has been revised to report the Nagelkerke R² value, indicating the proportion of variance in maternal undernutrition explained by the model. We further acknowledge that a substantial proportion of the variance remains unexplained, suggesting the influence of unmeasured factors.

The 75% of undernutrition in Mogadishu is really alarming and this may be attributed to an unmeasured exposure including the ongoing humanitarian crisis and displacement… We fully agree with the reviewer. The Discussion has been expanded to highlight the role of prolonged humanitarian crises, urban poverty, and the high concentration of internally displaced persons (IDPs) in Mogadishu as plausible contributors to the extremely high prevalence of maternal undernutrition observed in this study. We also acknowledge displacement status as an important unmeasured variable and recommend its inclusion in future research.

What possibly explains why attainment of tertiary education was a risk factor for undernutrition among pregnant women? The Discussion has been strengthened to provide a detailed contextual explanation. We now discuss how tertiary education in Somalia does not necessarily translate into stable employment or improved income for women, particularly in Mogadishu, where unemployment, informal labor, and occupational stress may override the expected nutritional benefits of education.

Similarly, previous contraceptive use associated positively with undernutrition. What accounts for this? And are there similar results in the literature? We thank the reviewer for this insightful comment. A new paragraph has been added to the Discussion explaining that previous contraceptive use may reflect cumulative reproductive stress, short birth intervals, or underlying socioeconomic vulnerability rather than a direct causal effect. We also note that similar associations have been reported in other low-resource settings and cite supporting literature accordingly.

Attachment Submitted filename: Point-by-point Reviewers Response.docx

# Peer review: Decision Letter 1

**DOI:** 10.1371/journal.pone.0347187.r003

**Authors:** Helen Howard

27 Apr 2026

<div>PONE-D-25-46984R1

Prevalence of Undernutrition and Its Associated Factors Among Pregnant Women Attending Antenatal Care Service in Public Hospitals in Mogadishu, Somalia

PLOS One

Dear Dr. Tahlil,

Thank you for submitting your manuscript to PLOS ONE. After careful consideration, we feel that it has merit but does not fully meet PLOS ONE’s publication criteria as it currently stands. Therefore, we invite you to submit a revised version of the manuscript that addresses the points raised during the review process.

Please submit your revised manuscript by Jun 08 2026 11:59PM. If you will need more time than this to complete your revisions, please reply to this message or contact the journal office at plosone@plos.org. When you're ready to submit your revision, log on to https://www.editorialmanager.com/pone/ and select the 'Submissions Needing Revision' folder to locate your manuscript file.

Please include the following items when submitting your revised manuscript:

- A letter that responds to each point raised by the academic editor and reviewer(s). You should upload this letter as a separate file labeled 'Response to Reviewers'.
- A marked-up copy of your manuscript that highlights changes made to the original version. You should upload this as a separate file labeled 'Revised Manuscript with Track Changes'.
- An unmarked version of your revised paper without tracked changes. You should upload this as a separate file labeled 'Manuscript'.

If you would like to make changes to your financial disclosure, please include your updated statement in your cover letter. Guidelines for resubmitting your figure files are available below the reviewer comments at the end of this letter.

If applicable, we recommend that you deposit your laboratory protocols in protocols.io to enhance the reproducibility of your results. Protocols.io assigns your protocol its own identifier (DOI) so that it can be cited independently in the future. For instructions see: https://journals.plos.org/plosone/s/submission-guidelines#loc-laboratory-protocols. Additionally, PLOS ONE offers an option for publishing peer-reviewed Lab Protocol articles, which describe protocols hosted on protocols.io. Read more information on sharing protocols at https://plos.org/protocols?utm_medium=editorial-email&utm_source=authorletters&utm_campaign=protocols.

As the corresponding author, your ORCID iD is verified in the submission system and will appear in the published article. PLOS supports the use of ORCID, and we encourage all coauthors to register for an ORCID iD and use it as well. Please encourage your coauthors to verify their ORCID iD within the submission system before final acceptance, as unverified ORCID iDs will not appear in the published article. *Only*  the individual author can complete the verification step; PLOS staff *cannot*  verify ORCID iDs on behalf of authors.

We look forward to receiving your revised manuscript.

Kind regards,

Helen Howard

Staff Editor

PLOS One

Journal Requirements:

If the reviewer comments include a recommendation to cite specific previously published works, please review and evaluate these publications to determine whether they are relevant and should be cited. There is no requirement to cite these works unless the editor has indicated otherwise.

[Note: HTML markup is below. Please do not edit.]

Reviewers' comments:

Reviewer's Responses to Questions

**Comments to the Author**

1. If the authors have adequately addressed your comments raised in a previous round of review and you feel that this manuscript is now acceptable for publication, you may indicate that here to bypass the “Comments to the Author” section, enter your conflict of interest statement in the “Confidential to Editor” section, and submit your "Accept" recommendation.

Reviewer #1: (No Response)

Reviewer #2: (No Response)

**********

2. Is the manuscript technically sound, and do the data support the conclusions?

The manuscript must describe a technically sound piece of scientific research with data that supports the conclusions. Experiments must have been conducted rigorously, with appropriate controls, replication, and sample sizes. The conclusions must be drawn appropriately based on the data presented.

Reviewer #1: Yes

Reviewer #2: Partly

**********

3. Has the statistical analysis been performed appropriately and rigorously?

Reviewer #1: Yes

Reviewer #2: No

**********

4. Have the authors made all data underlying the findings in their manuscript fully available?

The PLOS Data policy requires authors to make all data underlying the findings described in their manuscript fully available without restriction, with rare exception (please refer to the Data Availability Statement in the manuscript PDF file). The data should be provided as part of the manuscript or its supporting information, or deposited to a public repository. For example, in addition to summary statistics, the data points behind means, medians and variance measures should be available. If there are restrictions on publicly sharing data—e.g. participant privacy or use of data from a third party—those must be specified.

Reviewer #1: Yes

Reviewer #2: No

**********

5. Is the manuscript presented in an intelligible fashion and written in standard English?

PLOS ONE does not copyedit accepted manuscripts, so the language in submitted articles must be clear, correct, and unambiguous. Any typographical or grammatical errors should be corrected at revision, so please note any specific errors here.

Reviewer #1: Yes

Reviewer #2: Yes

**********

6. Review Comments to the Author

Please use the space provided to explain your answers to the questions above. You may also include additional comments for the author, including concerns about dual publication, research ethics, or publication ethics. (Please upload your review as an attachment if it exceeds 20,000 characters)

Reviewer #1: Main Concerns

Although most of my earlier concerns have been addressed, there are still a few that should be attended to make the paper publishable.

METHODS

Assessment of dietary diversity, and food security

i. Household Food Insecurity Access Scale (HFIAS): the authors should provide the reference that gives details of how this was actually measured?

ii. The explanation given for classifying dietary intake patterns of pregnant women as either adequate or inadequate is still not clear. According to the authors, food items consumed were grouped according to standard food groups (what does “standard” refer to here? how many food groups were involved). The authors also categorized. dietary intake as “adequate” if at least one food item from a given food group was consumed during the recall period and “inadequate” if none was consumed. So, if a woman was classified as adequate for a particular food group but inadequate for another food group, how then did the authors describe the dietary intake patterns of this woman? Let me just mention that there is a difference between food variety and dietary diversity. Dietary diversity and food variety are distinct nutritional concepts, often used to measure diet quality, with dietary diversity counting different food groups consumed while food variety counts the total number of individual food items.

Food Variety Score (FVS): This is a simple count of the individual food items consumed over a specific period (e.g., 24 hours). For instance, eating an apple, a banana, and a pear would count as a variety of three items.

Dietary Diversity Score (DDS): This measures the number of food groups represented in a diet. Using the same example, an apple, a banana, and a pear would only count as one for dietary diversity because they all belong to the "fruit" group.

STUDY RESULTS

The results presented in Table 4 could be improved to show the overall dietary diversity of pregnant women. Dietary adequacy should not be based only on individual food groups but all the food groups. For example, the minimum dietary diversity for women (MDD-W) is achieved if the woman consumes five or more of the 10 defined food groups. Studies indicate that dietary diversity is often a more effective predictor of overall nutrient adequacy because consuming foods from multiple groups provides a better balance of energy, protein, and minerals than simply eating many different items within a single group.

Reviewer #2: Abstract

1. The reported prevalence of undernutrition (75.7%) is exceptionally high and requires contextual qualification. Please briefly acknowledge in the Abstract that this estimate is based on MUAC (<23 cm) and a facility-based sample, which may limit comparability with national estimates.

2. (Results section): Several adjusted odds ratios (e.g., private business AOR = 9.79; second trimester AOR = 8.59) appear unusually large. Consider adding a short cautionary phrase indicating that these associations should be interpreted carefully.

Introduction

3. While the background is comprehensive, the distinction between BMI-based national estimates and MUAC-based assessment should be more clearly and concisely articulated earlier in the section to strengthen the study rationale.

4. The justification for focusing on an urban, facility-based population is improved but could be further sharpened by explicitly stating why ANC attendees in Mogadishu represent a high-risk subgroup.

Methods (Study Design): The facility-based cross-sectional design should be more explicitly acknowledged as limiting generalizability, particularly given that women not attending ANC are excluded.

5. (Sampling): Additional clarity is needed regarding how the sampling interval (k) was operationalized in each hospital. Providing a brief example would improve reproducibility.

6. (Anthropometry): The use of MUAC <23 cm as a cutoff requires stronger justification with citation of WHO or emergency nutrition guidelines, as this threshold critically influences the prevalence estimate.

7. (Dietary Assessment): The classification of dietary intake as “adequate” based on consumption of at least one item per food group over 24 hours appears overly simplistic. This should be reframed as “food group consumption” rather than adequacy.

8. (Statistical Analysis): While VIF was used to assess multicollinearity, additional details on model-building strategy (e.g., theoretical vs stepwise selection) would strengthen transparency. The choice of p<0.25 for inclusion in multivariable analysis should be justified with a citation or brief rationale.

Results

9. (Prevalence): The very high prevalence (75.7%) requires further descriptive support. Reporting the mean and distribution (e.g., SD or percentiles) of MUAC would help readers assess plausibility.

10. (Table 5): Some categories (e.g., tertiary education, n=53) are relatively small, yet show strong associations (AOR = 3.43). Please comment on the stability of these estimates.

11. (Regression findings): The magnitude of certain associations (e.g., second trimester AOR = 8.59; private business AOR = 9.79) raises concern for residual confounding or model instability. This should be acknowledged.

12. Interpretive language is occasionally used (e.g., explanation of education findings). Interpretation should be reserved for the Discussion section.

Discussion

13. (Prevalence): While comparisons with other countries are extensive, the discussion should more critically address why the prevalence in this study is substantially higher than both national (15%) and regional estimates.

14. (Education finding): The explanation provided for tertiary education being associated with undernutrition is speculative. It would be helpful to explicitly acknowledge alternative explanations such as confounding or measurement bias.

15. (Trimester effect): The very strong association between trimester and undernutrition requires cautious interpretation. Consider discussing whether MUAC changes across pregnancy stages may influence this finding.

16. (Model performance): The reported Nagelkerke R² (~42%) is mentioned but not critically interpreted. Please clarify what proportion of variability remains unexplained and what key variables may be missing.

17. (Bias): Selection bias (facility-based sample), recall bias (24-hour dietary recall), and unmeasured confounders (e.g., displacement status) should be more explicitly discussed.

18. (Food insecurity): Given that over half of households were severely food insecure, the interaction between food insecurity and other predictors could be explored or at least acknowledged.

Conclusion

19. The conclusions are currently somewhat strong relative to the observational design. Statements implying causality should be softened (e.g., replace “determinants” with “associated factors”).

20. Policy recommendations are appropriate but should be framed as being based on associative evidence rather than causal inference.

21. Data Availability: The statement that data are “available upon reasonable request” does not meet PLOS ONE requirements. Please revise to include a public repository or provide a justified exception.

22. Overall clarity: The manuscript is generally well-structured, but minor language refinement would improve readability, particularly in the Results and Discussion sections.

**********

7. PLOS authors have the option to publish the peer review history of their article (what does this mean?). If published, this will include your full peer review and any attached files.

If you choose “no”, your identity will remain anonymous but your review may still be made public.

**Do you want your identity to be public for this peer review?**  For information about this choice, including consent withdrawal, please see our Privacy Policy.

Reviewer #1: **Yes:**  Mahama Saaka

Reviewer #2: **Yes:**  Abu Ansar Md Rizwan

**********

[NOTE: If reviewer comments were submitted as an attachment file, they will be attached to this email and accessible via the submission site. Please log into your account, locate the manuscript record, and check for the action link "View Attachments". If this link does not appear, there are no attachment files.]

To ensure your figures meet our technical requirements, please review our figure guidelines: https://journals.plos.org/plosone/s/figures

You may also use PLOS’s free figure tool, NAAS, to help you prepare publication quality figures: https://journals.plos.org/plosone/s/figures#loc-tools-for-figure-preparation.

NAAS will assess whether your figures meet our technical requirements by comparing each figure against our figure specifications.

# Author comment: Author response to Decision Letter 2

**DOI:** 10.1371/journal.pone.0347187.r004

29 Apr 2026

POINT-BY-POINT REVIEWER'S RESPONSE

Reviewer 1

Reviewer’s Comment Author’s Response

METHODS – Assessment of dietary diversity, and food security

i. Household Food Insecurity Access Scale (HFIAS): the authors should provide the reference that gives details of how this was actually measured? Thank you for this important comment. We have revised the Methods section to clearly describe the HFIAS measurement and included the appropriate reference (FANTA guideline). Specifically, we now state that HFIAS consists of nine standardized occurrence questions covering three domains (anxiety, food quality, and food intake), with frequency-of-occurrence responses used to classify households into four categories (food secure, mildly, moderately, and severely food insecure).

ii. The explanation given for classifying dietary intake patterns of pregnant women as either adequate or inadequate is still not clear. According to the authors, food items consumed were grouped according to standard food groups (what does “standard” refer to here? how many food groups were involved). We appreciate this clarification request. The Methods section has been revised to explicitly define “standard food groups” as the ten food groups specified by the Minimum Dietary Diversity for Women (MDD-W) indicator. These ten groups are now clearly listed in the manuscript to ensure transparency and reproducibility.

The authors also categorized dietary intake as “adequate” if at least one food item from a given food group was consumed during the recall period and “inadequate” if none was consumed. Thank you for highlighting this ambiguity. We have revised the manuscript to clarify that consumption of individual food groups was used only to describe dietary patterns, not to determine overall dietary adequacy.

So, if a woman was classified as adequate for a particular food group but inadequate for another food group, how then did the authors describe the dietary intake patterns of this woman? We have addressed this concern by clearly distinguishing between food group consumption and overall dietary adequacy. Specifically, we now state that overall dietary adequacy was determined using the Dietary Diversity Score (DDS), which summarizes the number of food groups consumed. Individual food group consumption is presented descriptively and does not determine overall adequacy.

Let me just mention that there is a difference between food variety and dietary diversity. We thank the reviewer for this important distinction. The Methods section has been revised to explicitly differentiate between food variety (number of individual food items consumed) and dietary diversity (number of food groups consumed). This clarification aligns with established nutritional assessment frameworks.

Food Variety Score (FVS): This is a simple count of the individual food items consumed over a specific period… We acknowledge this explanation and have incorporated the conceptual distinction into the manuscript. We clarify that our study used Dietary Diversity Score (DDS), not Food Variety Score (FVS), to assess overall diet quality.

Dietary Diversity Score (DDS): This measures the number of food groups represented in a diet… We have revised the Methods section to clearly describe DDS calculation based on the ten MDD-W food groups. We also specified the cut-off point, where consumption of ≥5 food groups indicate adequate dietary diversity, consistent with international guidelines.

STUDY RESULTS

The results presented in Table 4 could be improved to show the overall dietary diversity of pregnant women. Thank you for this suggestion. We have revised the Results section to prioritize Dietary Diversity Score (DDS) as the main indicator of overall dietary adequacy. The DDS findings are now presented first before describing individual food group consumption.

Dietary adequacy should not be based only on individual food groups but all the food groups. We agree and have addressed this by clarifying that overall dietary adequacy is determined using DDS (based on all food groups combined), rather than individual food groups. Individual food group consumption is now clearly presented as descriptive information only.

For example, the minimum dietary diversity for women (MDD-W) is achieved if the woman consumes five or more of the 10 defined food groups. This recommendation has been incorporated into the manuscript. We now explicitly state that adequate dietary diversity is defined as consumption of at least five out of the ten MDD-W food groups.

Studies indicate that dietary diversity is often a more effective predictor of overall nutrient adequacy… We appreciate this insight and have strengthened the manuscript by emphasizing that DDS is a proxy indicator of micronutrient adequacy and overall diet quality, consistent with established literature. Additionally, the Results section has been revised to reflect this emphasis.

Reviewer 2

Reviewer’s Comment Author’s Response

1. The reported prevalence of undernutrition (75.7%) is exceptionally high and requires contextual qualification. Please briefly acknowledge in the Abstract that this estimate is based on MUAC (<23 cm) and a facility-based sample, which may limit comparability with national estimates. We thank the reviewer for this important observation. The Abstract has been revised to clearly indicate that the reported prevalence is based on MUAC (<23 cm) and a facility-based sample, which may limit comparability with national estimates derived from BMI-based population surveys.

2. (Results section): Several adjusted odds ratios (e.g., private business AOR = 9.79; second trimester AOR = 8.59) appear unusually large. Consider adding a short cautionary phrase indicating that these associations should be interpreted carefully. We appreciate this comment. A cautionary statement has been added to the Results section indicating that large effect sizes should be interpreted with caution due to potential residual confounding, small subgroup sizes, or model instability.

3. While the background is comprehensive, the distinction between BMI-based national estimates and MUAC-based assessment should be more clearly and concisely articulated earlier in the section to strengthen the study rationale. We thank the reviewer for this suggestion. The Introduction has been revised to more clearly and concisely distinguish between BMI-based national estimates and MUAC-based assessment earlier in the section.

4. The justification for focusing on an urban, facility-based population is improved but could be further sharpened by explicitly stating why ANC attendees in Mogadishu represent a high-risk subgroup. We appreciate this comment. The Introduction has been strengthened by explicitly stating that ANC attendees in Mogadishu represent a high-risk subgroup due to urban poverty, displacement, and food insecurity.

Methods (Study Design): The facility-based cross-sectional design should be more explicitly acknowledged as limiting generalizability, particularly given that women not attending ANC are excluded. We thank the reviewer for this important point. The limitation of the facility-based cross-sectional design in restricting generalizability has been explicitly acknowledged in the Discussion section.

5. (Sampling): Additional clarity is needed regarding how the sampling interval (k) was operationalized in each hospital. Providing a brief example would improve reproducibility. We appreciate this suggestion. A detailed example illustrating how the sampling interval (k) was calculated and applied has been added to improve clarity and reproducibility.

6. (Anthropometry): The use of MUAC <23 cm as a cutoff requires stronger justification with citation of WHO or emergency nutrition guidelines, as this threshold critically influences the prevalence estimate. We thank the reviewer. The manuscript has been revised to include stronger justification for the MUAC <23 cm cutoff, supported by WHO and emergency nutrition guidelines.

7. (Dietary Assessment): The classification of dietary intake as “adequate” based on consumption of at least one item per food group over 24 hours appears overly simplistic. This should be reframed as “food group consumption” rather than adequacy. We appreciate this important comment. The terminology has been revised throughout the manuscript to reflect “food group consumption (Yes/No)” rather than adequacy, and overall dietary adequacy is now determined using DDS.

8. (Statistical Analysis): While VIF was used to assess multicollinearity, additional details on model-building strategy (e.g., theoretical vs stepwise selection) would strengthen transparency. The choice of p<0.25 for inclusion in multivariable analysis should be justified with a citation or brief rationale. We thank the reviewer. The model-building strategy has been clarified to include both statistical and theoretical considerations, and justification for the use of p<0.25 has been provided.

9. (Prevalence): The very high prevalence (75.7%) requires further descriptive support. Reporting the mean and distribution (e.g., SD or percentiles) of MUAC would help readers assess plausibility. We appreciate this comment. The mean and standard deviation of MUAC have been included to provide additional descriptive support for the reported prevalence.

10. (Table 5): Some categories (e.g., tertiary education, n=53) are relatively small, yet show strong associations (AOR = 3.43). Please comment on the stability of these estimates. We thank the reviewer. A statement has been added acknowledging that small subgroup sizes may affect the stability and precision of the estimates.

11. (Regression findings): The magnitude of certain associations (e.g., second trimester AOR = 8.59; private business AOR = 9.79) raises concern for residual confounding or model instability. This should be acknowledged. We appreciate this comment. A statement has been added acknowledging that large effect sizes may reflect residual confounding or model instability.

12. Interpretive language is occasionally used (e.g., explanation of education findings). Interpretation should be reserved for the Discussion section. We thank the reviewer. Interpretive language has been removed from the Results section and appropriately relocated to the Discussion section.

13. (Prevalence): While comparisons with other countries are extensive, the discussion should more critically address why the prevalence in this study is substantially higher than both national (15%) and regional estimates. We appreciate this comment. The Discussion has been strengthened to critically explain the higher prevalence based on methodological and contextual differences.

14. (Education finding): The explanation provided for tertiary education being associated with undernutrition is speculative. It would be helpful to explicitly acknowledge alternative explanations such as confounding or measurement bias. We thank the reviewer. The explanation has been revised to reduce speculation and to acknowledge potential confounding and measurement bias.

15. (Trimester effect): The very strong association between trimester and undernutrition requires cautious interpretation. Consider discussing whether MUAC changes across pregnancy stages may influence this finding. We appreciate this suggestion. The Discussion now includes a cautious interpretation and consideration of physiological changes affecting MUAC during pregnancy.

16. (Model performance): The reported Nagelkerke R² (~42%) is mentioned but not critically interpreted. Please clarify what proportion of variability remains unexplained and what key variables may be missing. We thank the reviewer. The Discussion has been expanded to interpret the unexplained variance and identify potential missing variables.

17. (Bias): Selection bias (facility-based sample), recall bias (24-hour dietary recall), and unmeasured confounders (e.g., displacement status) should be more explicitly discussed. We appreciate this comment. The limitations section has been strengthened to explicitly address these biases.

18. (Food insecurity): Given that over half of households were severely food insecure, the interaction between food insecurity and other predictors could be explored or at least acknowledged. We thank the reviewer. A statement has been added acknowledging potential interactions and recommending further research.

19. The conclusions are currently somewhat strong relative to the observational design. Statements implying causality should be softened (e.g., replace “determinants” with “associated factors”). We appreciate this comment. The Conclusion has been revised to remove causal language and replace it with associative terminology.

20. Policy recommendations are appropriate but should be framed as being based on associative evidence rather than causal inference. We thank the reviewer. Policy recommendations have been reframed to reflect that they are based on observed associations.

21. Data Availability: The statement that data are “available upon reasonable request” does not meet PLOS ONE requirements. Please revise to include a public repository or provide a justified exception. We appreciate this comment. The data availability statement has been revised to reflect ethical restrictions and IRB approval requirements.

22. Overall clarity: The manuscript is generally well-structured, but minor language refinement would improve readability, particularly in the Results and Discussion sections. We thank the reviewer for this helpful suggestion. The manuscript has been carefully revised to improve clarity, coherence, and readability, particularly in the Results and Discussion sections.

Attachment Submitted filename: Response to Reviewers.docx

# Peer review: Decision Letter 2

**DOI:** 10.1371/journal.pone.0347187.r005

**Authors:** Olutosin Ademola Otekunrin

25 May 2026

PONE-D-25-46984R2

Prevalence of Undernutrition and Its Associated Factors Among Pregnant Women Attending Antenatal Care Service in Public Hospitals in Mogadishu, Somalia

PLOS One

Dear Dr. Tahlil,

Thank you for submitting your manuscript to PLOS ONE. After careful consideration, we feel that it has merit but does not fully meet PLOS ONE’s publication criteria as it currently stands. Therefore, we invite you to submit a revised version of the manuscript that addresses the points raised during the review process.

==============================

ACADEMIC EDITOR: Kindly address the comments of the reviewer before this manuscript can move to the next stage. Thank you./>

==============================

Please submit your revised manuscript by Jul 09 2026 11:59PM. If you will need more time than this to complete your revisions, please reply to this message or contact the journal office at plosone@plos.org. When you're ready to submit your revision, log on to https://www.editorialmanager.com/pone/ and select the 'Submissions Needing Revision' folder to locate your manuscript file.

Please include the following items when submitting your revised manuscript:

- A letter that responds to each point raised by the academic editor and reviewer(s). You should upload this letter as a separate file labeled 'Response to Reviewers'.
- A marked-up copy of your manuscript that highlights changes made to the original version. You should upload this as a separate file labeled 'Revised Manuscript with Track Changes'.
- An unmarked version of your revised paper without tracked changes. You should upload this as a separate file labeled 'Manuscript'.

If you would like to make changes to your financial disclosure, please include your updated statement in your cover letter. Guidelines for resubmitting your figure files are available below the reviewer comments at the end of this letter.

If applicable, we recommend that you deposit your laboratory protocols in protocols.io to enhance the reproducibility of your results. Protocols.io assigns your protocol its own identifier (DOI) so that it can be cited independently in the future. For instructions see: https://journals.plos.org/plosone/s/submission-guidelines#loc-laboratory-protocols. Additionally, PLOS ONE offers an option for publishing peer-reviewed Lab Protocol articles, which describe protocols hosted on protocols.io. Read more information on sharing protocols at https://plos.org/protocols?utm_medium=editorial-email&utm_source=authorletters&utm_campaign=protocols.

As the corresponding author, your ORCID iD is verified in the submission system and will appear in the published article. PLOS supports the use of ORCID, and we encourage all coauthors to register for an ORCID iD and use it as well. Please encourage your coauthors to verify their ORCID iD within the submission system before final acceptance, as unverified ORCID iDs will not appear in the published article. *Only*  the individual author can complete the verification step; PLOS staff *cannot*  verify ORCID iDs on behalf of authors.

We look forward to receiving your revised manuscript.

Kind regards,

Olutosin Ademola Otekunrin, PhD

Academic Editor

PLOS One

Journal Requirements:

If the reviewer comments include a recommendation to cite specific previously published works, please review and evaluate these publications to determine whether they are relevant and should be cited. There is no requirement to cite these works unless the editor has indicated otherwise.

Please review your reference list to ensure that it is complete and correct. If you have cited papers that have been retracted, please include the rationale for doing so in the manuscript text, or remove these references and replace them with relevant current references. Any changes to the reference list should be mentioned in the rebuttal letter that accompanies your revised manuscript. If you need to cite a retracted article, indicate the article’s retracted status in the References list and also include a citation and full reference for the retraction notice.

[Note: HTML markup is below. Please do not edit.]

Reviewers' comments:

Reviewer's Responses to Questions

-->Comments to the Author

1. If the authors have adequately addressed your comments raised in a previous round of review and you feel that this manuscript is now acceptable for publication, you may indicate that here to bypass the “Comments to the Author” section, enter your conflict of interest statement in the “Confidential to Editor” section, and submit your "Accept" recommendation.

Reviewer #1: All comments have been addressed

**********

2. Is the manuscript technically sound, and do the data support the conclusions?

The manuscript must describe a technically sound piece of scientific research with data that supports the conclusions. Experiments must have been conducted rigorously, with appropriate controls, replication, and sample sizes. The conclusions must be drawn appropriately based on the data presented.

Reviewer #1: Yes

**********

3. Has the statistical analysis been performed appropriately and rigorously?

Reviewer #1: Yes

**********

4. Have the authors made all data underlying the findings in their manuscript fully available?

The PLOS Data policy requires authors to make all data underlying the findings described in their manuscript fully available without restriction, with rare exception (please refer to the Data Availability Statement in the manuscript PDF file). The data should be provided as part of the manuscript or its supporting information, or deposited to a public repository. For example, in addition to summary statistics, the data points behind means, medians and variance measures should be available. If there are restrictions on publicly sharing data—e.g. participant privacy or use of data from a third party—those must be specified.

Reviewer #1: Yes

**********

5. Is the manuscript presented in an intelligible fashion and written in standard English?

PLOS ONE does not copyedit accepted manuscripts, so the language in submitted articles must be clear, correct, and unambiguous. Any typographical or grammatical errors should be corrected at revision, so please note any specific errors here.

Reviewer #1: Yes

**********

6. Review Comments to the Author

Please use the space provided to explain your answers to the questions above. You may also include additional comments for the author, including concerns about dual publication, research ethics, or publication ethics. (Please upload your review as an attachment if it exceeds 20,000 characters)

Reviewer #1: All my concerns have been adequately addressed to make the paper publishable as the paper has significant findings.

**********

7. PLOS authors have the option to publish the peer review history of their article (what does this mean?). If published, this will include your full peer review and any attached files.

If you choose “no”, your identity will remain anonymous but your review may still be made public.

Do you want your identity to be public for this peer review? For information about this choice, including consent withdrawal, please see our Privacy Policy.

Reviewer #1: Yes: Dr, Mahama Saaka

**********

[NOTE: If reviewer comments were submitted as an attachment file, they will be attached to this email and accessible via the submission site. Please log into your account, locate the manuscript record, and check for the action link "View Attachments". If this link does not appear, there are no attachment files.]

To ensure your figures meet our technical requirements, please review our figure guidelines: https://journals.plos.org/plosone/s/figures

You may also use PLOS’s free figure tool, NAAS, to help you prepare publication quality figures: https://journals.plos.org/plosone/s/figures#loc-tools-for-figure-preparation.

NAAS will assess whether your figures meet our technical requirements by comparing each figure against our figure specifications.

# Author comment: Author response to Decision Letter 3

**DOI:** 10.1371/journal.pone.0347187.r006

10 Jun 2026

POINT-BY-POINT REVIEWER'S RESPONSE

Reviewer/Editor Comment Author Response

Thank you for providing the Data Availability Statement. We note that your underlying data are restricted for ethical or legal reasons. PLOS journals' data policy does not allow authors to be the sole contact for access to restricted data. Please provide contact information for a non-author institutional point of contact who can field data inquiries from fellow researchers and update the Data Availability Statement accordingly. Thank you for this important observation. We have revised the Data Availability Statement to comply with PLOS ONE's data-sharing requirements. Specifically, we have replaced the corresponding author as the sole contact for data access and included a non-author institutional point of contact through the Institutional Review Board (IRB) of Zamzam University of Science and Technology. Researchers seeking access to the restricted dataset may now submit requests directly to the IRB, which will review requests and determine eligibility for data access in accordance with ethical approval requirements and participant confidentiality protections.

# Peer review: Decision Letter 3

**DOI:** 10.1371/journal.pone.0347187.r007

**Authors:** Olutosin Ademola Otekunrin

18 Jun 2026

Prevalence of Undernutrition and Its Associated Factors Among Pregnant Women Attending Antenatal Care Service in Public Hospitals in Mogadishu, Somalia

PONE-D-25-46984R3

Dear Dr. Tahlil,

We’re pleased to inform you that your manuscript has been judged scientifically suitable for publication and will be formally accepted for publication once it meets all outstanding technical requirements.

Within one week, you’ll receive an e-mail detailing the required amendments. When these have been addressed, you’ll receive a formal acceptance letter and your manuscript will be scheduled for publication.

An invoice will be generated when your article is formally accepted. Please note, if your institution has a publishing partnership with PLOS and your article meets the relevant criteria, all or part of your publication costs will be covered. Please make sure your user information is up-to-date by logging into Editorial Manager at Editorial Manager® and clicking the ‘Update My Information' link at the top of the page. For questions related to billing, please contact billing support.

If your institution or institutions have a press office, please notify them about your upcoming paper to help maximize its impact. If they’ll be preparing press materials, please inform our press team as soon as possible -- no later than 48 hours after receiving the formal acceptance. Your manuscript will remain under strict press embargo until 2 pm Eastern Time on the date of publication. For more information, please contact onepress@plos.org.

Kind regards,

Olutosin Ademola Otekunrin, PhD

Academic Editor

PLOS One

Additional Editor Comments (optional):

Reviewers' comments:

Reviewer's Responses to Questions

**Comments to the Author**

1. If the authors have adequately addressed your comments raised in a previous round of review and you feel that this manuscript is now acceptable for publication, you may indicate that here to bypass the “Comments to the Author” section, enter your conflict of interest statement in the “Confidential to Editor” section, and submit your "Accept" recommendation.

Reviewer #1: All comments have been addressed

**********

2. Is the manuscript technically sound, and do the data support the conclusions?

The manuscript must describe a technically sound piece of scientific research with data that supports the conclusions. Experiments must have been conducted rigorously, with appropriate controls, replication, and sample sizes. The conclusions must be drawn appropriately based on the data presented.

Reviewer #1: Yes

**********

3. Has the statistical analysis been performed appropriately and rigorously?

Reviewer #1: Yes

**********

4. Have the authors made all data underlying the findings in their manuscript fully available?

The PLOS Data policy requires authors to make all data underlying the findings described in their manuscript fully available without restriction, with rare exception (please refer to the Data Availability Statement in the manuscript PDF file). The data should be provided as part of the manuscript or its supporting information, or deposited to a public repository. For example, in addition to summary statistics, the data points behind means, medians and variance measures should be available. If there are restrictions on publicly sharing data—e.g. participant privacy or use of data from a third party—those must be specified.

Reviewer #1: Yes

**********

5. Is the manuscript presented in an intelligible fashion and written in standard English?

PLOS ONE does not copyedit accepted manuscripts, so the language in submitted articles must be clear, correct, and unambiguous. Any typographical or grammatical errors should be corrected at revision, so please note any specific errors here.

Reviewer #1: Yes

**********

6. Review Comments to the Author

Please use the space provided to explain your answers to the questions above. You may also include additional comments for the author, including concerns about dual publication, research ethics, or publication ethics. (Please upload your review as an attachment if it exceeds 20,000 characters)

Reviewer #1: My earlier concerns have been addressed and the paper is now recommended to be publishable in the journal..

**********

7. PLOS authors have the option to publish the peer review history of their article (what does this mean?). If published, this will include your full peer review and any attached files.

If you choose “no”, your identity will remain anonymous but your review may still be made public.

**Do you want your identity to be public for this peer review?**  For information about this choice, including consent withdrawal, please see our Privacy Policy.

Reviewer #1: **Yes:**  Mahama Saaka

**********

# Editor report: Acceptance letter

**DOI:** 10.1371/journal.pone.0347187.r008

**Authors:** Olutosin Ademola Otekunrin

PONE-D-25-46984R3

PLOS One

Dear Dr. Tahlil,

I'm pleased to inform you that your manuscript has been deemed suitable for publication in PLOS One. Congratulations! Your manuscript is now being handed over to our production team.

At this stage, our production department will prepare your paper for publication. This includes ensuring the following:

* All references, tables, and figures are properly cited

* All relevant supporting information is included in the manuscript submission,

* There are no issues that prevent the paper from being properly typeset

You will receive further instructions from the production team, including instructions on how to review your proof when it is ready. Please keep in mind that we are working through a large volume of accepted articles, so please give us a few days to review your paper and let you know the next and final steps.

Lastly, if your institution or institutions have a press office, please let them know about your upcoming paper now to help maximize its impact. If they'll be preparing press materials, please inform our press team within the next 48 hours. Your manuscript will remain under strict press embargo until 2 pm Eastern Time on the date of publication. For more information, please contact onepress@plos.org.

You will receive an invoice from PLOS for your publication fee after your manuscript has reached the completed accept phase. If you receive an email requesting payment before acceptance or for any other service, this may be a phishing scheme. Learn how to identify phishing emails and protect your accounts at https://explore.plos.org/phishing.

If we can help with anything else, please email us at customercare@plos.org.

Thank you for submitting your work to PLOS ONE and supporting open access.

Kind regards,

PLOS ONE Editorial Office Staff

on behalf of

Dr. Olutosin Ademola Otekunrin

Academic Editor

PLOS One
